# Acute Copper Exposure Induces Immunotoxicity and Metabolic Reprogramming in Rainbow Trout (*Oncorhynchus mykiss*) Muscle via FoxO/mTOR/PPAR Pathways

**DOI:** 10.3390/biology15211913

**Published:** 2026-10-22

**Authors:** Shangqi Zhao, Jinqiang Quan, Deji Gao, Huinan Song, Mengyu Duan, Youchao Lin, Yujia Sun, Jiang Zheng

**Affiliations:** 1 Fisheries College, Jimei University, Xiamen 361021, China; 202421065023@jmu.edu.cn (S.Z.);; 2 College of Animal Science and Technology, Gansu Agricultural University, Lanzhou 730070, China; quanjq@gsau.edu.cn

**Keywords:** rainbow trout, copper immunotoxicity, TNF-α, FoxO/mTOR/PPAR pathways, multi-omics, inflammatory response

## Abstract

Copper sulfate (CuSO_4_) is widely used in aquaculture for disease prevention and control, which poses significant potential risks to fish health and welfare. This study investigated the response mechanisms of immune-related molecules in the muscle tissue of rainbow trout (*Oncorhynchus mykiss*) following acute sublethal copper concentrations and during the recovery period. Rainbow trout were exposed to nominal Cu^2+^ concentrations of 0, 75, 150, and 300 μg/L for 4 d, followed by a 2 day depuration period. Integrated biochemical assays, transcriptomics and metabolomics were applied to muscle tissue, focusing on immune relevant pathways. Copper exposure significantly inhibited acetylcholinesterase and Ca^2+^-ATPase activities, indicating neurotoxicity, and induced oxidative stress, as evidenced by altered antioxidant enzyme activities and increased lipid peroxidation. It is worth noting that, during the stress induction process, the pro-inflammatory cytokine TNF-α and genes associated with lipid metabolism (*Srebp1*, *FAS*) were continuously up-regulated, and remained slightly higher than in the control group even during the recovery period, leading to the accumulation of saturated fatty acids and inflammatory signaling; this indicates persistent dysregulation of transcription. Muscle copper content paradoxically decreased while total protein increased, suggesting active metal efflux and metabolic reprogramming. Integrated multi-omics identified MYH4 and SCD1 as key nodes, and KEGG and O2PLS analyses showed that copper-induced immunotoxicity was associated with coordinated dysregulation of genes enriched in FoxO, mTOR and PPAR signaling pathways—central regulators of inflammation, protein homeostasis and lipid metabolism. These pathway-level findings are correlative and require causal validation. These findings suggest that short-term exposure to acute sublethal copper concentrations is associated with persistent immunotoxic transcriptional alterations in rainbow trout muscle, characterized by sustained inflammatory signaling and metabolic disruption during the short recovery period. The identified pathways and targets (TNF α, MYH4, SCD1, FoxO/mTOR/PPAR) provide candidate biomarkers for assessing copper-induced immunotoxicity and contribute to understanding the immune risks of copper pollution in aquatic ecosystems.

## Simple Summary

Copper sulfate is widely used to control diseases in fish farming, but excessive copper exposure may harm fish. How brief exposure affects the natural defenses and chemical processes in fish muscle, and whether these effects continue after exposure ends, remains unclear. We investigated these responses in rainbow trout exposed to elevated copper levels for four days, followed by two days in clean water. We measured muscle protein activity and chemical composition, together with the activity of genes. Compared with unexposed fish, exposed fish showed increased damage from harmful chemical reactions, reduced activity of proteins supporting nerve signaling and muscle function, and altered fat and energy processing. Signals linked to inflammation, the body’s response to injury or stress, also increased. Although no fish died, some changes remained after two days in clean water, indicating incomplete recovery within this short period. These findings show that brief copper exposure can disrupt muscle health even without causing death. The results could help improve monitoring of copper exposure and fish recovery, guide more careful use of copper treatments, and support fish welfare and sustainable fish farming.

## 1. Background

Copper (Cu) is an essential trace mineral element required for the growth and reproduction of aquatic animals. At trace levels, Cu serves as a structural component of specific proteins or functions as a cofactor for enzymes critical to the antioxidant system and mitochondrial respiratory chain [1]. Numerous studies have demonstrated that Cu exposure can elicit significant homeostatic and metabolic responses in aquatic animals, which may lead to alterations in key physiological processes, including oxygen consumption [2], ammonia excretion [3], ionic and osmotic regulation [4], and acid-base regulation [5].

However, copper (Cu) can also function as a chemical pollutant released into the environment through industrial effluents, port operations, and domestic sewage [6]. At elevated concentrations, copper ions exert toxic effects on aquatic animals [7]. For example, in zebrafish (*Danio rerio*) muscle tissue, exposure to copper nanoparticles (nano-Cu) suppresses RAD51 gene expression. This suppression impairs DNA repair via the homologous recombination pathway and exacerbates metabolic dysfunction [8].

Copper concentrations in natural waters are typically low, ranging from 0.0008 to 0.001 mg L^−1^ [9]. However, anthropogenic activities, including industrial and municipal discharges, mining and metallurgical activities, and urban runoff, are recognized sources of copper to aquatic environments. Copper concentrations in waters are highly site-specific and depend strongly on water chemistry and proximity to point sources [9,10]. In aquaculture, the use of copper sulfate has also been studied as a water disinfection measure in freshwater recirculating aquaculture systems. Furthermore, a concentration of 0.5–0.7 mg/L CuSO_4_ (corresponding to a copper ion concentration of approximately 200–280 µg/L) is commonly used in aquaculture to treat parasitic infections in fish and to suppress or eliminate outbreaks of harmful algae, including cyanobacteria [11,12]. Chronic or sublethal exposure to copper can result in its accumulation within fish tissues, notably the brain, gills, gut, kidneys, and liver. Subsequent metabolic processes may further facilitate accumulation in additional tissues [13].

Rainbow trout (*Oncorhynchus mykiss*) is a commercially significant cold-water freshwater fish species, valued by both consumers and producers for its nutritional quality and adaptability to high-density aquaculture. It has become one of the most extensively farmed and productive species of its kind in China. Concomitant with the expansion of rainbow trout farming in recent years, disease incidence has risen, leading to increased reliance on chemotherapeutants. Notably, the use of CuSO_4_ has become prevalent [14]. Muscle is the main edible tissue and the primary aquaculture product. Copper-induced alterations in muscle immune and metabolic pathways have direct implications for seafood quality, nutritional value, and food safety. Although CuSO_4_ is widely used in aquaculture, the immunotoxic effects of acute exposure to high concentrations of Cu^2+^ on the muscles of farmed rainbow trout remain unknown. In this study, we exposed rainbow trout to different concentrations of copper sulfate for four days, followed by a two-day depuration period in clean water. Through a combined transcriptomic and metabolomic analysis of muscle tissue, we investigated the molecular characteristics of copper-induced immunopathology. This study provides a mechanistic framework for understanding how short-term metal stress reprograms immune and metabolic pathways in farmed fish, which has direct implications for disease susceptibility and health management in aquaculture.

## 2. Materials and Methods

### 2.1. Experimental Design and Sample Collection

In this study, 600 full-sibling rainbow trout (average weight: 81.8 ± 0.55 g, average body length: 17.8 ± 1.52 cm) were randomly allocated to a control group (Group T, comprising three replicate tanks, each containing 50 fish; cylindrical tank dimensions: 2 m in diameter × 1.5 m in height, with a water capacity of 2000 L) and three experimental groups (Groups A, B and C; each comprising three replicate tanks, each containing 50 fish). The juvenile rainbow trout were initially acclimatized in an indoor recirculating aquaculture system. Copper sulfate pentahydrate (CuSO_4_·5H_2_O, AR, Sinopharm Chemical Reagents Co., Ltd., Beijing, China) was used as the copper source. Based on preliminary acute toxicity tests, the 96 h LC_50_ of Cu^2+^ for rainbow trout was 500 µg/L (0.5 mg/L). To assess sublethal immunotoxic effects under an acute challenge, nominal Cu^2+^ concentrations of 0 (T), 75 (A), 150 (B), and 300 (C) µg/L were used (the concentration of copper ions in the water was calculated and prepared accordingly; it was not measured in practice). The highest concentration (300 µg/L) corresponds to 60% of the 96 h LC_50_ and is designated as an acute sublethal challenge concentration, not a safe or NOEC level. The conventional safe range (0.01–0.1 LC_50_) would be 5–50 µg/L; therefore, the exposure concentrations used here represent an acute sublethal stress scenario rather than environmentally relevant chronic exposure. This was followed by a 2-day purification phase in copper-free culture water, bringing the total duration of the experiment to 6 days. Throughout the entire trial period, including the acclimatization phase, the fish were fed twice daily (at 8.00 am and 6.00 pm) at a rate of 2% of their body weight. No mortality occurred during the study. To maintain the target Cu^2+^ concentrations, 80% of the water in each tank was replaced daily with pre-prepared aquaculture water containing consistent levels of copper. Dissolved oxygen was maintained at 8.5 ± 1.05 mg/L by intermittent aeration. Water temperature and pH were maintained at 18.0 ± 0.5 °C and 7.5, respectively. Water hardness (expressed as CaCO_3_) was <200 mg/L; average concentration of dissolved organic carbon (DOC) was approximately 7.58 mg/L; nitrite was below 0.01 mg/L; ammonia nitrogen was below 0.1 mg/L.

Sampling occurred on exposure days 1, 2, 3, 4, and recovery days 1 and 2. Six fish per tank were anesthetized using 30 mg L^−1^ MS-222 (Sigma-Aldrich Co., St. Louis, MO, USA). Fish were then rapidly dissected; liver, head kidney, spleen, gills, brain, intestine, and dorsal white muscle tissues were excised. Tissues were rinsed with ice-cold physiological saline (0.9% NaCl), immediately flash-frozen in liquid nitrogen, and subsequently stored at −80 °C for further analysis. The fish sampling method is as follows: six fish were taken from each tank, totaling 18 fish per treatment group. For biochemical analysis and RT-qPCR testing, n = 3 biological replicates were used for each treatment group at each time point. Each biological replicate consisted of a pooled muscle tissue sample from three fish randomly selected from the same replicate tank (tank-level replicate). The remaining fish in each tank were allocated for other analyses [e.g., muscle copper content, total protein, histology].

The sampling times were coded as exposure days D1–D4 and recovery days R1–R2. For omics analyses, T4, B4, and B6 represent the control at day 4, exposure to 150 μg/L Cu^2+^ for 4 days, and exposure to 150 μg/L Cu^2+^ for 4 days followed by 2 days of depuration, respectively. T4 vs. B4 assesses the acute exposure effect, B4 vs. B6 assesses the recovery effect, and T4 vs. B6 assesses the residual effect after the short recovery period.

### 2.2. Enzyme Activity and Gene Expression Tests

Fresh dorsal white muscle samples from experimental groups (three biological replicates per group per timepoint) were assayed to quantify enzymatic activities and biochemical markers. Measurements included: total superoxide dismutase (T-SOD), glutathione reductase (GR), lactate dehydrogenase (LDH), catalase (CAT), acetylcholinesterase (AChE), succinate dehydrogenase (SDH), Ca^2+^-ATPase (Ca^2+^-ATP), total antioxidant capacity (T-AOC), glutathione peroxidase (GSH-Px) activity and malondialdehyde (MDA) content. Assays were performed using commercial kits (Jiancheng Bioengineering Institute, Nanjing, China) strictly adhering to manufacturer protocols. Absorbance values were determined with a multimode microplate reader (Thermo Scientific™ Varioskan™ LUX, Model FI01620; Vantaa, Finland).

Total RNA was extracted from tissue samples using TRIzol™ reagent (Takara Bio Inc., Dalian, China). mRNA expression levels of target genes were quantified via reverse transcription quantitative polymerase chain reaction (RT-qPCR). Complementary DNA (cDNA) synthesis was performed using the BioRT High Sensitive cDNA First Strand Synthesis Kit (BioFlux, Hangzhou, China). RT-qPCR primer sequences for all target genes are shown in Table 1. Fresh dorsal white muscle samples from experimental groups (three biological replicates per group per timepoint) were analyzed to quantify the mRNA expression of genes functionally associated with the following: hsp70, hsp90, TNF-α, LP1, cat-1α, Aoc, fas, srebp1, TOR.

Statistical analysis of biochemical parameters and RT-qPCR data is described in Section 2.9.

### 2.3. Tests for the Accumulation of Copper and Protein in Rainbow Trout Muscle

Total Cu was determined by AAS after validated acid digestion. A total of 1 g of muscle tissue was digested in 5 mL of concentrated HNO3 overnight. Tissue samples were then carefully filtered in Watmann grade 1 filter paper (Whatman International Ltd., Maidstone, UK). The filtrate was diluted to 100 mL in a volumetric flask mixed with 5 mL concentrated HCl. The mixture was then placed in a fume bath for 15 min. After heating, the samples were cooled and filtered by Millipore filter paper. Finally, the filtrates were digested by a proportional mixture of concentrated HNO3-HClO4 and filtered again in Watmann filter paper (grade 42) (Whatman International Ltd., Maidstone, UK) before determination of heavy metal concentration in AAS.

Crude protein content was determined using the Kjeldahl method as follows. A certain amount of muscle tissue sample (usually 0.5–2 g) was weighed and placed into a Kessler flask (Jiangsu Huida Medical Devices Co., Ltd., Jiangsu, Yancheng, China) with 10 mL of concentrated sulfuric acid (Sinopharm Chemical Reagents Co., Ltd., Beijing, China). A total of 0.5 g of copper sulfate (Sinopharm Chemical Reagents Co., Ltd., Beijing, China) and 5 g of potassium sulfate (Sinopharm Chemical Reagents Co., Ltd., Beijing, China) were added as catalysts. The Kjeldahl flask was heated on the digester until the sample was completely decomposed and the solution turned clear blue-green, and the digester was cooled. The cooled digest was transferred to a distillation unit, a 40% sodium hydroxide solution (Sinopharm Chemical Reagents Co., Ltd., Beijing, China) was added to neutralize the acidic digest, and ammonia gas was released. The distillation is started, and the distilled ammonia is absorbed with a boric acid solution (usually 2%, Sinopharm Chemical Reagents Co., Ltd., Beijing, China). A few drops of mixed indicator are added, and the color of the absorbed solution turns green. The boric acid (Sinopharm Chemical Reagents Co., Ltd., Beijing, China) solution after absorption of ammonia gas was titrated with a standard acid solution, such as 0.1 N hydrochloric acid (Sinopharm Chemical Reagents Co., Ltd., Beijing, China) or sulfuric acid (Sinopharm Chemical Reagents Co., Ltd., Beijing, China), until the color changed from green to gray-red, according to the standard volume calculation of nitrogen content of acid solution, so as to calculate the crude protein content. Muscle dry matter was determined by drying samples at 105 °C to constant weight.

### 2.4. RNA Extraction, Strand-Specific Library Construction and Sequencing

Total RNA was extracted using Trizol reagent kit (Invitrogen, Carlsbad, CA, USA) according to the manufacturer’s protocol. RNA quality was assessed on an Agilent 2100 Bioanalyzer (Agilent Technologies, Palo Alto, CA, USA) and checked using RNase free agarose gel electrophoresis. After total RNA was extracted, eukaryotic mRNA was enriched by Oligo(dT) beads. Then the enriched mRNA was fragmented into short fragments using fragmentation buffer and reversely transcribed into cDNA by using NEBNext Ultra RNA Library Prep Kit for Illumina (NEB#7530, New England Biolabs, Ipswich, MA, USA). The purified double-stranded cDNA fragments were end repaired, A base added, and ligated to Illumina sequencing adapters. The ligation reaction was purified with the AMPure XP Beads (1.0X). And polymerase chain reaction (PCR) amplified. The resulting cDNA library was sequenced using Illumina Novaseq6000 by Gene Denovo Biotechnology Co. (Guangzhou, China).

### 2.5. Bioinformatics Analysis of Transcriptome

Based on the 2020 update of the rainbow trout reference genome USDA_OmykA_1.1 (https://www.ncbi.nlm.nih.gov/datasets/genome/GCF_013265735.2/, accessed on 21 September 2024) an index of the reference genome was created, and paired-end clean sequences were mapped to the reference genome using HISAT2. The genes/transcripts with the parameter of false discovery rate (FDR) below 0.05 and absolute fold change ≥2 were considered differentially expressed genes/transcripts. The bioinformatics analysis, alignment with reference genome, quantification of gene abundance, differentially expressed genes (DEGs), GO and KEGG enrichment analysis and trend analysis are presented in Supplementary File S1.

### 2.6. Metabolomic Assays

#### 2.6.1. Sample Collection and Preparation

The rainbow trout muscle tissue was quickly frozen in liquid nitrogen immediately after dissection. Then the tissue was cut on dry ice (~80 mg) into an Eppendorf tube (2 mL, Sinopharm Chemical Reagents Co., Ltd., Beijing, China). The tissue samples with 200 μL of H_2_O and five ceramic beads were homogenized using the homogenizer. A total of 800 μL methanol/acetonitrile (1:1, *v*/*v*) (Sinopharm Chemical Reagents Co., Ltd., Beijing, China)was added to homogenized solution for metabolite extraction. The mixture was centrifuged for 15 min (14,000× *g*, 4 °C). The supernatant was dried in a vacuum centrifuge. For LC-MS analysis, the samples were re-dissolved in 100 μL acetonitrile/water (1:1, *v*/*v*) solvent.

#### 2.6.2. LC-MS/MS Analysis

Analysis was performed using an UHPLC (1290 Infinity LC, Agilent Technologies) coupled to a quadrupole time-of-flight (AB Sciex TripleTOF 6600) at Shengong Biotechnology Co., Ltd., Shanghai, China.

For HILIC separation, samples were analyzed using a 2.1 mm × 100 mm ACQUIY UPLC BEH 1.7 μm column (Waters, Wexford, Ireland). In both ESI positive and negative modes, the mobile phase contained A = 25 mM ammonium acetate and 25 mM ammonium hydroxide in water and B = acetonitrile. The gradient was 85% B for 1 min and was linearly reduced to 65% in 11 min, and then was reduced to 40% in 0.1 min and kept for 4 min, and then increased to 85% in 0.1 min, with a 5 min re-equilibration period employed.

For RPLC separation, a 2.1 mm × 100 mm ACQUIY UPLC HSS T3 1.8 μm column (Waters Corporation, Milford, MA, USA) was used. In ESI positive mode, the mobile phase contained A = water with 0.1% formic acid and B = acetonitrile with 0.1% formic acid; and in ESI negative mode, the mobile phase contained A = 0.5 mM ammonium fluoride in water and B = acetonitrile. The gradient was 1%B for 1.5 min and was linearly increased to 99% in 11.5 min and kept for 3.5 min. Then it was reduced to 1% in 0.1 min, and a 3.4 min re-equilibration period was employed. The gradients were at a flow rate of 0.3mL/min, and the column temperatures were kept constant at 25 °C. A 2 μL aliquot of each sample was injected.

The ESI source conditions were set as follows: Ion Source Gas1 (Gas1) as 60, Ion Source Gas2 (Gas2) as 60, curtain gas (CUR) as 30, source temperature 600 °C, IonSpray Voltage Floating (ISVF) ± 5500 V. In MS only acquisition, the instrument was set to acquire over the *m*/*z* range 60–1000 Da, and the accumulation time for the TOF MS scan was set at 0.20 s/spectra. In auto MS/MS acquisition, the instrument was set to acquire over the *m*/*z* range 25–1000 Da, and the accumulation time for product ion scan was set at 0.05 s/spectra. The product ion scan is acquired using information-dependent acquisition (IDA) with high-sensitivity mode selected. The parameters were set as follows: the collision energy (CE) was fixed at 35 V with ± 15 eV; declustering potential (DP), 60 V (+) and −60 V (−); exclude isotopes within 4 Da; candidate ions to monitor per cycle: 10.

#### 2.6.3. Data Processing

The raw MS data were converted to MzXML files using ProteoWizard MSConvert (Version 3.0.8789) before importing into freely available XCMS software (Version 3.23). For peak picking, the following parameters were used: centWave *m*/*z* = 10 ppm, peakwidth = c (10, 60), prefilter = c (10, 100). For peak grouping, bw = 5, mzwid = 0.025, and minfrac = 0.5 were used. CAMERA (Collection of Algorithms of MEtabolite pRofile Annotation) was used for annotation of isotopes and adducts. In the extracted ion features, only the variables having more than 50% of the nonzero measurement values in at least one group were kept. Compound identification of metabolites was performed by comparing of accuracy *m*/*z* value (<10 ppm), and MS/MS spectra with an in-house database established with available authentic standards. The missing data were filled by KNN (K-Nearest Neighbor) method, and the extreme values were deleted. Finally, the total peak area of the data was normalized to ensure the parallelism between samples and metabolites.

### 2.7. Bioinformatics Analysis of Metabolome

A variable importance in projection (VIP) score of the (O)PLS model was applied to rank the metabolites that best distinguished between two groups. Metabolomic data were log-transformed and Pareto-scaled before multivariate analysis. PCA was performed as an unsupervised exploratory method to visualize the overall variance among T4, B4, and B6. No significance test was applied to PCA score plots. The threshold of VIP was set to 1. In addition, *p*-value < 0.05 and VIP ≥ 1 were considered differential metabolites between two groups. The data quality control, cluster analysis, multivariate statistical analysis, differential metabolites analysis, pathway analysis and trend analysis are presented in Supplementary File S2.

### 2.8. Metabolome–Transcriptome Association Analysis

#### 2.8.1. Pathway Model

KEGG pathway maps are the linking of genomic or transcriptomic contents of genes to chemical structures of endogenous molecules, thus providing a method to perform integration analysis of genes and metabolites. All differentially expressed genes and metabolites in this study were mapped to the KEGG pathway database to obtain their links in metabolic pathways.

#### 2.8.2. O2PLS Model

In order to integrate the transcriptomic and metabolomic data, we performed a Twoway Orthogonal PLS (O2PLS) analysis. This method decomposes the variation present in the two data matrices into three parts: the joint variation between the two datasets, the orthogonal variation that is unique to each dataset and noise. The model assumes that some latent variables are responsible for the variation in the joint and orthogonal parts. O2PLS models were calculated using the OmicsPLS package of R (Version 2.0.2). To determine the optimal number of components, the proposed alternative cross-validation procedure was utilized. The best models were used for integration analysis.

#### 2.8.3. Pearson Model

Pearson correlation coefficients were calculated for metabolome and transcriptome data integration. Gene and metabolite pairs were ranked in the descending order of absolute correlation coefficients. The top 50 genes and metabolites were selected for heatmap analysis using pheatmap packages in R project. Additionally, the top 250 pairs of genes and metabolites (with the absolute Pearson correlation > 0.5) were applied for metabolite transcript network analysis using igraph packages in R project.

### 2.9. Statistical Analysis

Statistical analysis was performed using SPSS 19.0. Data are expressed as the mean ± standard deviation (SD). Differences among the four experimental groups (T, A, B, C) were assessed by one-way ANOVA followed by Tukey’s HSD post hoc test. The relative expression of target gene transcripts was calculated using the comparative threshold cycle method (2^−ΔΔCt^). A value of *p* < 0.05 was considered statistically significant.

## 3. Results

### 3.1. Changes in Biochemical Indices and Gene Expression During Copper Sulfate Treatment and Recovery Periods

**Figure 1:**
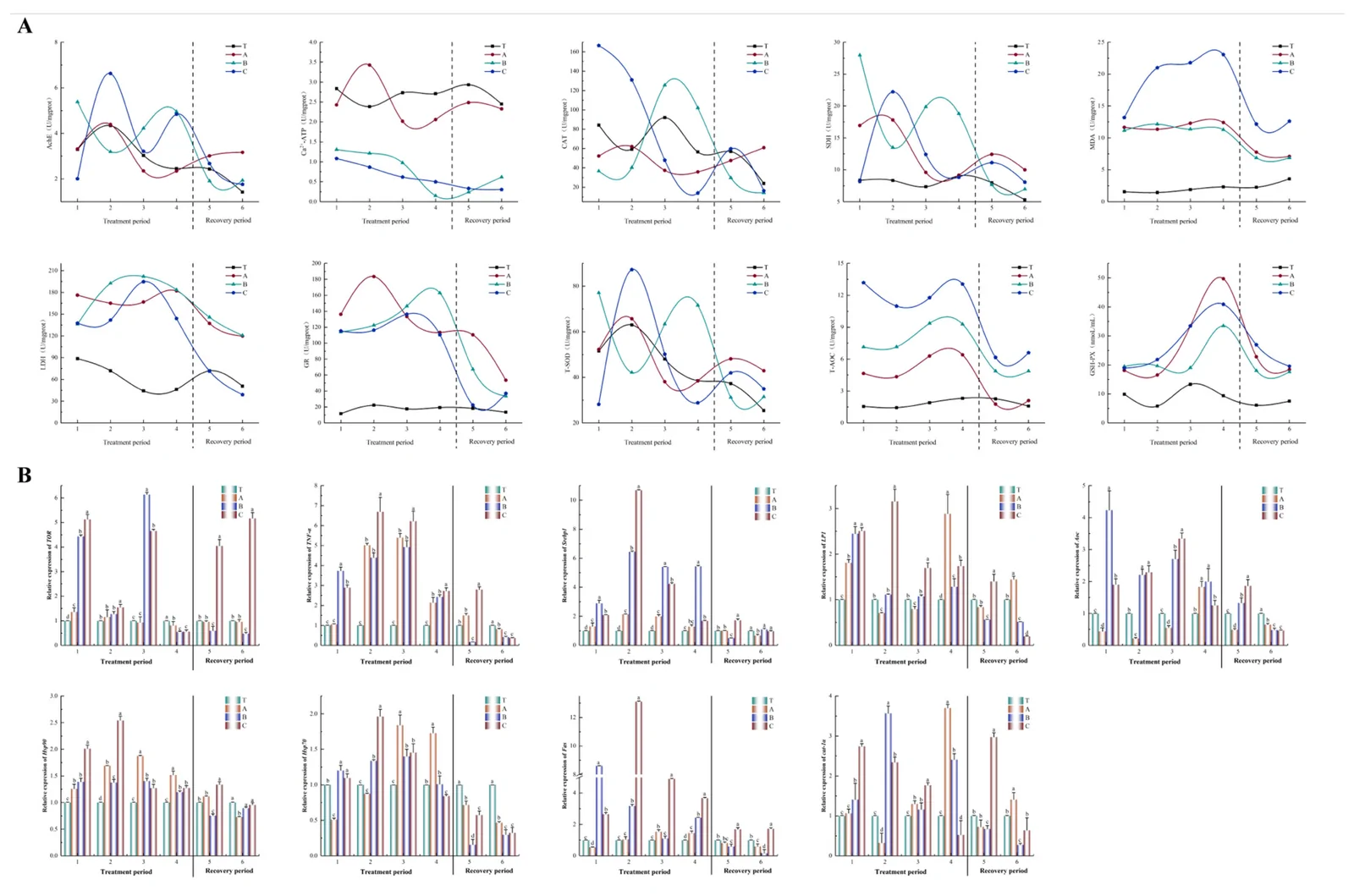
(**A**): Muscle enzyme activities and oxidative stress markers in rainbow trout during exposure and recovery. Enzymatic antioxidants (CAT, T-SOD, GSH-Px, GR), total antioxidant capacity (T-AOC), lipid peroxidation marker (MDA), and metabolic/tissue damage markers (LDH, SDH) are shown. Different letters indicate significant differences among groups (Tukey’s HSD, *p* < 0.05); (**B**): variation trend of muscle gene expression in rainbow trout during stress and recovery periods, different lowercase letters indicate significant differences within a group (*p* < 0.05).

Ten biochemical parameters associated with muscle antioxidant capacity, immune response, and meat quality were analyzed (Figure 1A). Acetylcholinesterase (AChE) activity exhibited an irregular pattern during the exposure phase compared to the control group (Group T), with particularly inconsistent responses observed under varying Cu^2+^ concentrations. Furthermore, AChE activity was significantly suppressed throughout the depuration phase. CAT activity exhibited marked time-dependent fluctuations across the groups, with Groups B and C showing greater variations during the treatment period, whilst levels generally tended to decline or fall back during the recovery period. T-SOD activity in all groups showed marked fluctuations, with periodic peaks during the treatment period and an overall decline during the recovery period; Group A, however, maintained a relatively high level throughout the recovery period. Ca^2+^-ATPase activity decreased significantly (*p* < 0.05) with increasing Cu^2+^ concentration, with the most pronounced reductions occurring in groups B and C. SDH activity fluctuated across all groups: in Group B, it was high at the start of the treatment period but then declined steadily; in Group A, it rebounded during the recovery period; whilst in Group T, the overall variation was minimal. MDA levels in Group T remained consistently low, whereas in Groups A, B and C, they were generally high throughout the treatment period but fell significantly upon entering the recovery period. LDH activity in Group T declined continuously before rising slightly during the recovery phase; Groups A and B showed an overall downward trend, whilst Group C initially increased during the treatment period before declining rapidly. GR activity in Group T remained consistently low and stable, whereas Groups A, B and C maintained relatively high levels during the treatment period, exhibiting phased changes, and generally declined during the recovery phase. T-AOC levels in Group T remained consistently low and stable, whilst in Groups A, B and C they fluctuated during the treatment period before declining in the later stages, and generally remained at low levels during the recovery period. GSH-Px activity in all groups exhibited a fluctuating trend of initially rising and then falling during the treatment period; Groups A and C reached relatively high levels in the later stages of treatment, before falling markedly during the recovery period.

The mRNA expression levels of nine genes functionally associated with muscle antioxidant capacity, immune response, and meat quality regulation TOR, TNF-α, Srebp1, LP1, Aoc, Hsp90, Hsp70, FAS, and cat-1α were quantified (Figure 1B). TOR expression generally followed a pattern of initially increasing and then fluctuating during the treatment period, with Groups B and C reaching higher levels in the middle phase, followed by an overall decline during the recovery phase. TNF-α expression increased significantly during the treatment period, reaching a high level in the middle phase, before falling back slightly and declining further during the recovery phase. Srebp1 expression fluctuated markedly during the treatment period; Group C exhibited a significant peak in Phase 2, followed by a rapid decline, and remained at a generally low level throughout the recovery phase. LP1 expression generally showed a phased increase during the treatment period, accompanied by marked differences between groups; some treatment groups reached higher levels in the middle phase, with a marked decline during the recovery period. AOC expression generally exhibited a fluctuating upward trend during the treatment period; Group B reached a high level in the early phase, whilst Groups A and C showed a more pronounced increase in the middle phase, with an overall decline during the recovery period. Hsp90 expression generally showed an initial increase followed by a decrease during the treatment period; Group C reached its highest level in Phase 2, whilst all groups tended towards lower levels during the recovery period. Hsp70 expression fluctuated markedly during the treatment period; Group A maintained a relatively high level in Phases 3–4, whereas Group C showed a gradual decline following an initial increase, with overall reduction during the recovery period. FAS expression levels rose markedly during the treatment period and exhibited periodic peaks; Group C reached its highest value in Phase 2, followed by an overall decline, and remained at a low level during the recovery period. cat-1α expression levels fluctuated significantly during the treatment period, with higher expression observed in Group B in the early stages and in Group A in the middle stages, followed by an overall decline; levels remained low throughout the recovery period.

The results of combined enzyme activity and gene expression were analyzed, and the day 4 control (T4), the addition of 150 µg/L treatment for 4 days (B4), and the addition of 150 µg/L recovery for 2 days (B6) were screened for subsequent analysis.

### 3.2. Accumulation of Copper and Protein in Rainbow Trout Muscle

**Figure 2:**
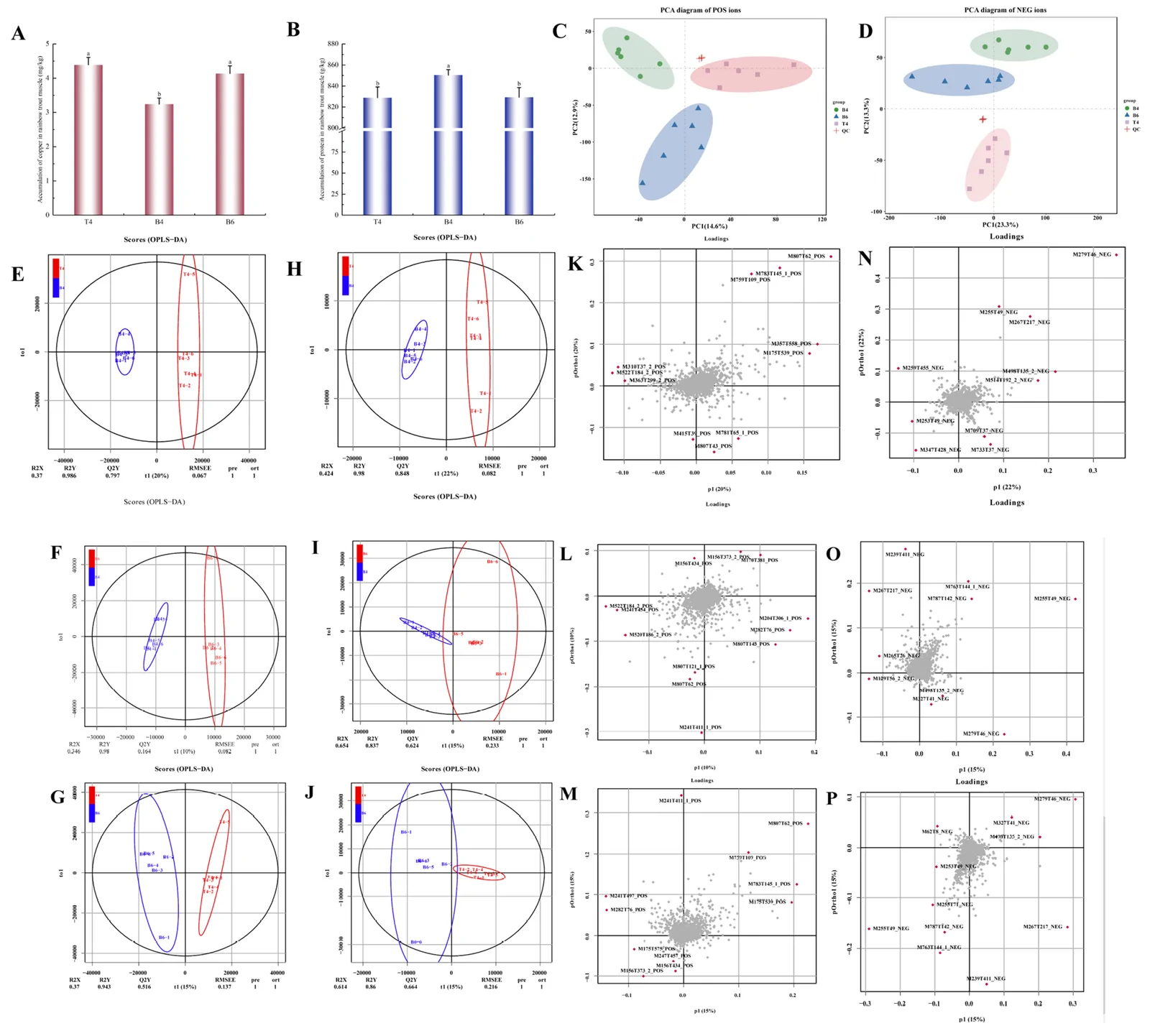
(**A**): Compared to the T4, copper content in rainbow trout muscle during stress and recovery periods, different lowercase letters indicate significant differences within a group (*p* < 0.05). (**B**): compared to the T4, protein content in rainbow trout muscle during stress and recovery periods, different lowercase letters indicate significant differences within a group (*p* < 0.05). (**C**): PCA results of POS ions; (**D**): NEG results of POS ions; (**E**): OPLS-DA analysis of POS ions in T4; (**F**): OPLS-DA analysis of POS ions in B4; (**G**): OPLS-DA analysis of POS ions in B6; (**H**): OPLS-DA analysis of NEG ions in T4; (**I**): OPLS-DA analysis of NEG ions in B4; (**J**): OPLS-DA analysis of NEG ions in B6; (**K**): the key POS ions in T4 vs. B4; (**L**): the key POS ions in B4 vs. B6; (**M**): the key POS ions in T4 vs. B6; (**N**): the key NEG ions in T4 vs. B4; (**O**): the key NEG ions in B4 vs. B6; (**P**): the key NEG ions in T4 vs. B6.

Analysis of copper ion (Cu^2+^) accumulation in dorsal white muscle tissue revealed significant differences between groups (Figure 2A). The B4 group (150 μg L^−1^ Cu^2+^ exposure for 4 days) exhibited temporarily significantly lower muscular Cu^2+^ concentrations than both the control group (T4) and depurated group (B6) (*p* < 0.05). Conversely, total protein content measurements (Figure 2B) demonstrated significantly higher levels in group B4 compared to T4 and B6 groups (*p* < 0.05).

### 3.3. Changes at the Transcriptome Level and Metabolome Level

Transcriptomic analysis was performed on dorsal white muscle tissue from nine rainbow trout, generating 56.2 Gb of raw sequencing data (Table 2). Following quality control, the average Q30 score was 95.5%, with a mean of 41.2 million high-quality reads per sample. Alignment to the reference genome (*Oncorhynchus mykiss* GCA_013265735.2) achieved 96.8% coverage, with 94.3% of reads mapped to exonic regions. Comparative genomic analysis identified 37,049 expressed genes, comprising 36,555 previously annotated genes and 494 novel transcripts.

Metabolomic profiling of dorsal white muscle tissue from 18 rainbow trout was conducted using liquid chromatography–tandem mass spectrometry (LC-MS/MS). Principal component analysis (PCA) of positive-ion (POS) and negative-ion (NEG) modes revealed distinct clustering patterns: each experimental group (T4, B4, B6) formed separate clusters with characteristic intra-group homogeneity (Figure 2C,D). This demonstrates that both acute copper ion (Cu^2+^) exposure (B4) and post-exposure depuration (B6) induced significant metabolic alterations relative to controls (T4). Orthogonal projections to latent structures–discriminant analysis (OPLS-DA) further confirmed group separation in pairwise comparisons (POS ions mode: Figure 2E–G; NEG ions mode: Figure 2H–J). All models exhibited significant inter-group discrimination (VIP > 1.0, *p* < 0.05), indicating that Cu^2+^ stress substantially modulates both cationic and anionic metabolite profiles in rainbow trout muscle.

Based on the high-resolution mass spectrometry (HRMS) detection technique, combined with public databases such as Mass Bank, Metlin, and MoNA, we detected a total of 12,300 POS ions. Of the detected features, 2571 positive-mode and 2086 negative-mode features were putatively annotated. Based on the OPLS-DA model loading diagram, we found 34 POS ions away from the coordinate origin among the identified ions, of which 11 were present in T4 vs. B4 (Figure 2K), 12 in B4 vs. B6 (Figure 2L), and 11 in T4 vs. B6 (Figure 2M). Similarly, 31 NEG ions were located away from the coordinate origin, with 10 present in T4 vs. B4 (Figure 2N), 10 in B4 vs. B6 (Figure 2O), and 11 in T4 vs. B6 (Figure 2P). This suggests that these POS/NEG ions away from the coordinate origin provide the most significant contribution to the differences in metabolite pattern changes between groups.

### 3.4. Trend Analysis of DEGs and Differential Metabolites

**Figure 3:**
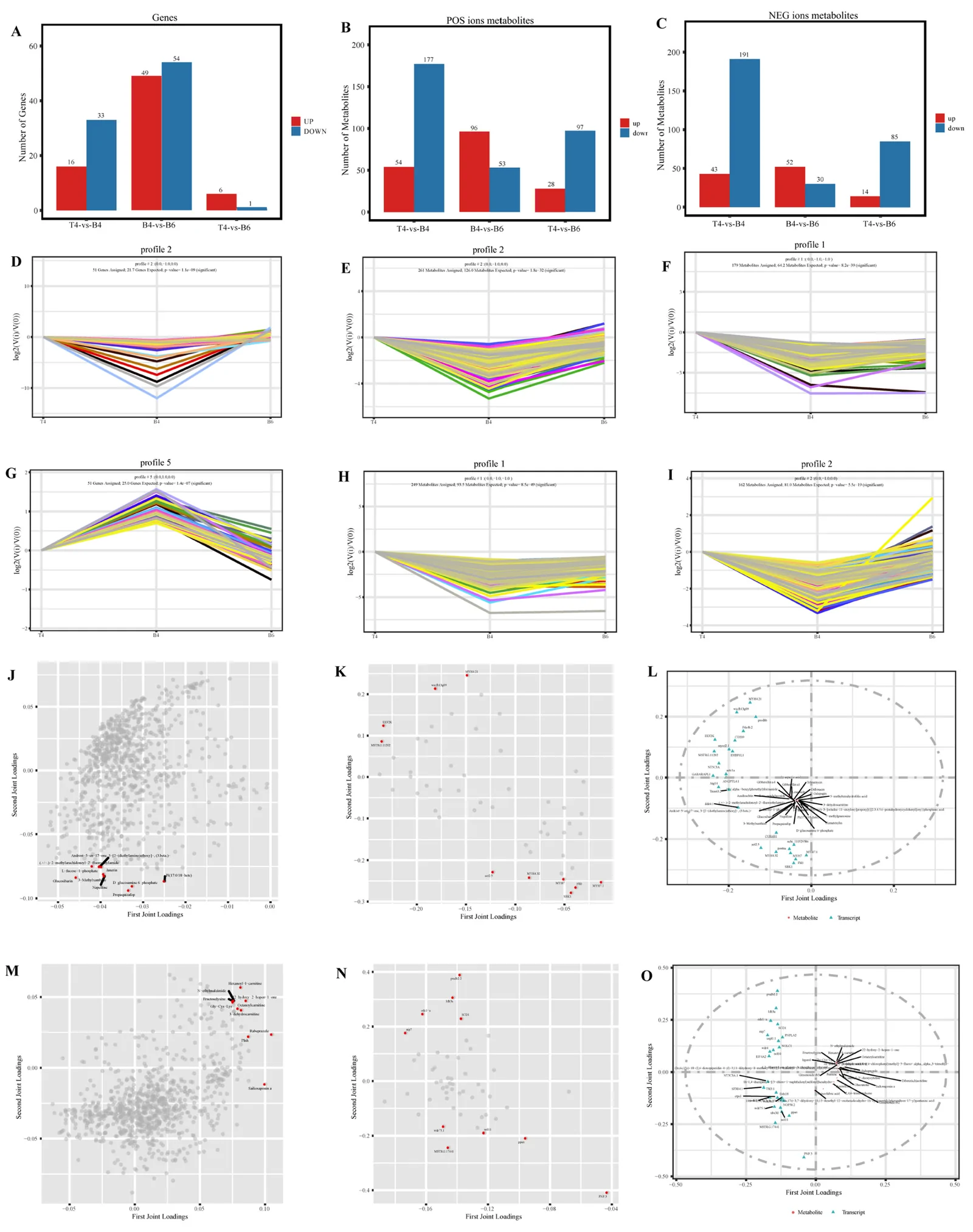
(**A**): The number of differentially expressed genes; (**B**): Differences in metabolites of POS ions; (**C**): differences in metabolites of NEG ions; (**D**,**G**): Profiles 2 and 5 of DEGs; (**E**,**H**): Profiles 2 and 1 of POS ion metabolites; (**F**,**I**): Profiles 1 and 2 of NEG ion metabolites; (**J**): the key POS ions of the joint analysis; (**K**): the key genes related to POS ions in the combined analysis; (**L**): joint analysis of key DEGs and POS ion metabolites; (**M**): the key NEG ions of the joint analysis; (**N**): the key genes related to NEG ions in the combined analysis; (**O**): joint analysis of key DEGs and NEG ion metabolites.

Differential gene expression analysis between groups T4, B4, and B6 identified significant transcriptomic changes (Figure 3A). Pairwise comparisons revealed the following: T4 vs. B4: 49 differentially expressed genes (DEGs; 16 upregulated, 33 downregulated), B4 vs. B6: 103 DEGs (49 upregulated, 54 downregulated), T4 vs. B6: 7 DEGs (six upregulated, one downregulated). Time-series clustering of DEGs across sequential timepoints (T4 → B4 → B6) identified 140 genes with significant expression trajectories. Profiles 2 and 5 demonstrated the most pronounced patterns (Figure 3D,G), each containing 51 DEGs. Profile 2: progressive downregulation during exposure (T4 → B4) followed by significant upregulation during depuration (B4 → B6); Profile 5: inverse pattern-sustained upregulation during exposure with subsequent downregulation during depuration.

Differential metabolites were identified by integrating Variable Importance in Projection (VIP) scores from orthogonal projections to latent structures–discriminant analysis (OPLS-DA) with univariate statistical significance (*p* < 0.05), applying thresholds of VIP ≥ 1 and *p* < 0.05. In positive-ion mode (POS): T4 vs. B4: 231 differential metabolites (55 upregulated, 177 downregulated), B4 vs. B6: 149 differential metabolites (96 upregulated, 53 downregulated), T4 vs. B6: 125 differential metabolites (28 upregulated, 97 downregulated) (Figure 3B). Temporal trend analysis of POS metabolites across T4 → B4 → B6 identified 779 features with significant trajectories. Profiles 2 (Figure 3E) and 1 (Figure 3H) demonstrated pronounced enrichment. Profile 2 (261 metabolites): progressive downregulation during exposure (T4 → B4) followed by significant upregulation during depuration (B4 → B6). Profile 1 (249 metabolites): sustained downregulation during exposure with stabilization during depuration.

Similarly, in negative-ion mode (NEG), differential metabolite counts were quantified (Figure 3C): T4 vs. B4: 234 differential metabolites (43 upregulated, 191 downregulated), B4 vs. B6: 82 differential metabolites (52 upregulated, 30 downregulated), T4 vs. B6: 99 differential metabolites (14 upregulated, 85 downregulated). Temporal trajectory analysis of NEG metabolites across T4 → B4 → B6 identified 519 features with significant expression patterns. Profiles 1 (Figure 3F) and 2 (Figure 3I) showed prominent enrichment. Profile 2 (162 metabolites): progressive downregulation during exposure followed by significant upregulation during depuration. Profile 1 (179 metabolites): sustained downregulation during exposure with stabilization during depuration.

### 3.5. Combined Analysis of Key DEGs and Differential Metabolites

To screen for interconnections between metabolites and genes, we plotted loadings for different variables for each of the variables in the joint section. The results showed that some POS ions (Figure 3J) and some genes (Figure 3K) played important roles in the whole trend process, such as COX6B1, ddit4, MYH7, FABP2, 3-Methylxanthine, 5-methyltetrahydrofolic acid, Hamamelitannin and so on (Figure 3L). Meanwhile, some NEG ions (Figure 3M) and some genes (Figure 3N) play important roles in the whole trend process, such as pnpla2, odc1-a, scd1, Solasodine, Peimine, Lycopsamine and so on (Figure 3O).

**Figure 4:**
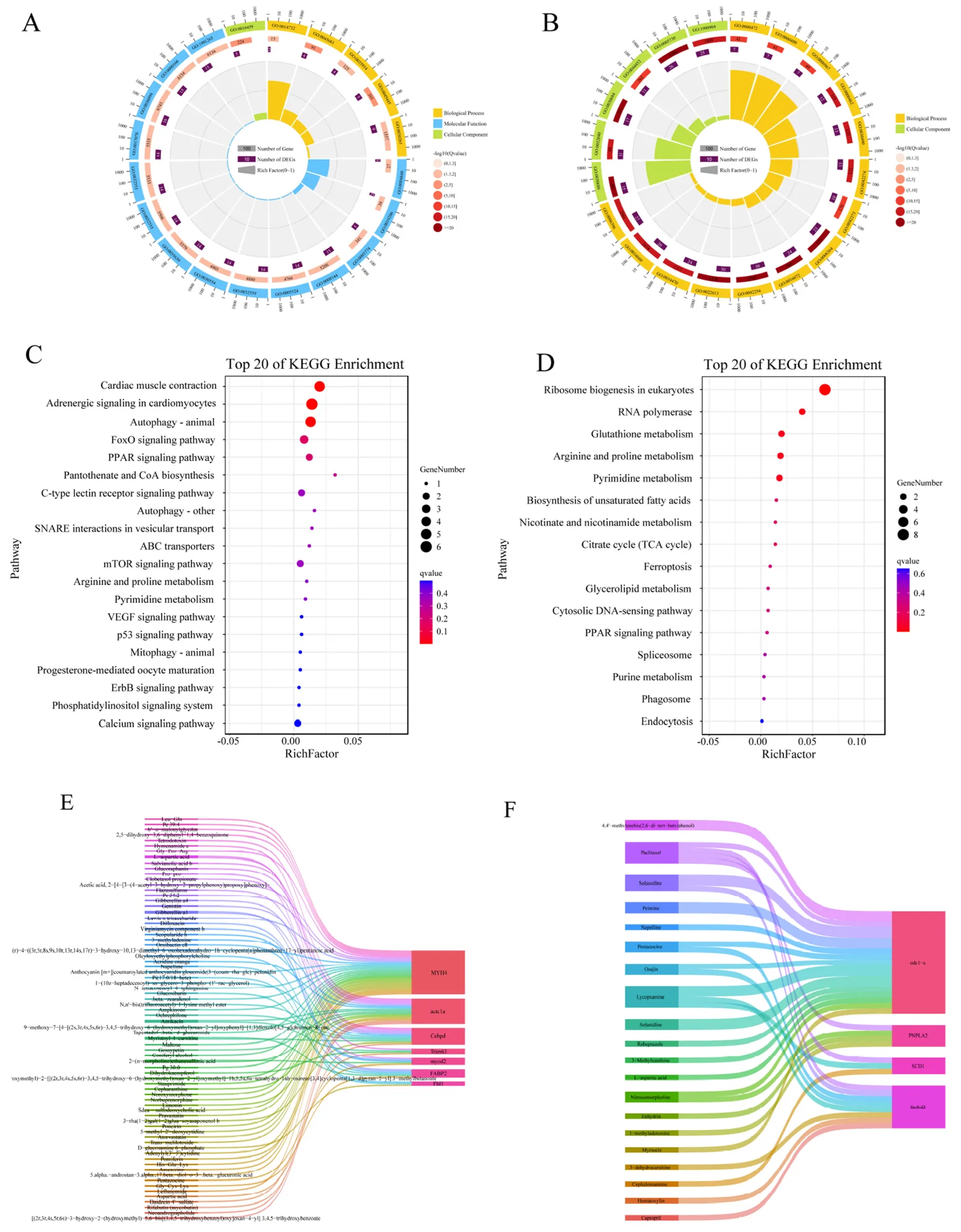
(**A**): GO terms for the key genes screened in Profile 2; (**B**): GO terms for the key genes screened in profile 5; (**C**): KEGG for the key genes screened in Profile 2; (**D**): KEGG for the key genes screened in Profile 5; (**E**): the relationship between key metabolites and gene-targeted regulation in Profile 2; (**F**): the relationship between key metabolites and gene-targeted regulation in Profile 5.

Based on the above results, we further performed GO and KEGG enrichment analyses for the key genes screened in profile 2 (Figure 4A) and profile 5 (Figure 4B). GO enrichment analysis of Profile 2 showed significant enrichment of myoblast differentiation (GO:0045445), myosin complex (GO:0016459), regulation of myoblast differentiation (GO:0045661), and skeletal muscle cell differentiation (GO:0035914), while in Profile 5, ribosome biogenesis (GO:0042254), rRNA metabolic process (GO:0016072), nucleolus (GO:0005730), ncRNA metabolic process (GO:0034660) and other processes were significantly enriched.

KEGG enrichment analysis of Profile 2 showed significant enrichment of the FoxO signaling pathway (ko04068), pantothenic acid and CoA biosynthesis (ko00770), the mTOR signaling pathway (ko04150), arginine and proline metabolism (ko00330), the ErbB signaling pathway (ko04012), and the calcium signaling pathway (ko04020). In Profile 5, glutathione metabolism (ko00480), arginine and proline metabolism (ko00330), pyrimidine metabolism (ko00240), biosynthesis of unsaturated fatty acids (ko01040), nicotinic acid and nicotinamide metabolism (ko00760), and the TCA cycle (ko00020) were significantly enriched. This suggests that these pathways play an important role in rainbow trout muscle tissue changes during copper ion stress as well as during recovery.

### 3.6. Functional Annotation and Analysis of Key Genes and Metabolites

Based on the results of DEGs, DEs and trend analyses, we finally screened out some key genes and metabolites involved in the regulation of muscle tissue growth and development and the regulation of meat quality in rainbow trout muscle tissues during 4 d of Cu-ion stress followed by 2 days of recovery, and carried out linkage analyses based on the correlation between the two. The results showed that in Profile 2 (Figure 4E), seven DEGs were significantly correlated with 74 DEs (correlation > 0.85), and some genes and metabolites related to cell growth and development and meat quality were screened out, such as MYH4, actela, Cebpd, Trim63, myod2, FABP2, and Fhl genes and Glucosibarin, Hymenamide c, L-aspartic acid, Salvianolic acid b, Clobetasol propionate, Flazasulfuron, etc. In Profile 5 (Figure 4F), four DEGs were significantly correlated with 20 DEs (correlation > 0.85), and some genes and metabolites related to cell growth and development and meat quality were screened, such as odc1-a, PNPLA2, SCD1, and fastkd2 genes and L-aspartic acld, 1-methyladenosine, 3-dehydrocarnitine, Enhydrin and so on.

## 4. Discussion

Copper (Cu) is an essential trace mineral that supports not only fundamental developmental processes in fish—such as hematopoiesis [15], osteogenesis [16], and neurodevelopment [17]—but also acts as a catalytic cofactor for key metalloenzymes including cytochrome c oxidase and Cu/Zn-superoxide dismutase [18]. Beyond these well-recognized roles, emerging evidence points to a critical function of copper in the development and functional maturation of the piscine immune system. Optimal copper availability has been shown to enhance growth performance [19,20,21], which in fish is intrinsically linked to the integrity of immune-competent tissues (e.g., the gut-associated lymphoid tissue and the head kidney). In aquaculture practice, copper sulfate (CuSO_4_) is routinely applied at 0.7 mg/L (providing 280 μg/L Cu^2+^) for antimicrobial treatment. However, our preliminary toxicity assessment established a 96 h median lethal concentration (LC_50_) of 500 μg/L Cu^2+^ for rainbow trout. Our preliminary 96 h LC_50_ for rainbow trout was 500 µg/L Cu^2+^. Therefore, the 300 µg/L treatment represents approximately 60% of the LC_50_ and should be regarded as an acute sublethal challenge concentration rather than a maximum safe operational threshold. A safe concentration or NOEC would be expected to lie within 5–50 µg/L (0.01–0.1 LC_50_). The 75–300 µg/L exposure range used here therefore models an acute sublethal exposure scenario relevant to aquaculture treatment or contaminated receiving waters, not pristine environmental conditions.

### 4.1. Integrated Physiological Responses to Copper Stress in Fish Muscle: Neurotoxicity, Calcium Imbalance, Metabolic Disruption and Inflammatory Activation

Acetylcholinesterase (AChE) is a key enzyme in neural transmission in fish; it terminates neural signals by hydrolyzing acetylcholine (ACh), thereby regulating locomotion, respiration and escape responses [22]. Our results reveal a ‘decline–rise–decline’ trajectory of AChE activity under copper exposure, suggesting the presence of compensatory neural regulatory mechanisms in rainbow trout muscle. The persistent inhibition of enzyme activity during the recovery period implies that acute stress may lead to neurotoxicity.

Sarco/endoplasmic reticulum Ca^2+^-ATPase (SERCA), essential for maintaining cytosolic calcium homeostasis, prevents calcium overload by actively sequestering Ca^2+^ into sarcoplasmic reticulum stores to regulate excitation–contraction coupling [23]. Copper exposure ≥150 μg/L significantly inhibited SERCA activity (75 μg/L caused functional disruption), triggering a pathological cascade: muscle calcium overload → metabolic energy depletion → tissue damage → behavioral abnormalities (e.g., swimming dysfunction). Absence of enzymatic recovery post-exposure confirms neuromuscular impairment, consistent with observed behavioral pathologies.

Succinate dehydrogenase (SDH), a mitochondrial complex II enzyme, facilitates ATP synthesis and modulates tricarboxylic acid (TCA) cycle intermediates (e.g., fumarate) to sustain locomotory and cardiac functions [24]. SDH activity was significantly suppressed (*p* < 0.05) in exposed groups versus controls and continued declining during depuration, indicating persistent dysregulation of energy metabolism and potential lipid metabolic disturbances.

Glutathione reductase (GR) regenerates reduced glutathione (GSH) from oxidized glutathione disulfide (GSSG) and is essential for sustaining GSH-Px-mediated peroxide detoxification [25]. In this study, GR activity was significantly elevated in exposed groups relative to the control, consistent with a compensatory activation of the glutathione antioxidant system under copper-induced oxidative stress.

The antioxidant defense system exhibited complex and non-monotonic responses to copper exposure. The activities of CAT and T-SOD did not increase uniformly; instead, CAT activity dropped sharply below control levels by days 3–4 in the higher-concentration groups, while T-SOD displayed a non-monotonic pattern across concentrations and time points. In contrast, GSH-Px and GR activities, together with T-AOC, were generally elevated relative to the control group during both exposure and depuration, suggesting a compensatory activation of glutathione-dependent and non-enzymatic antioxidant defenses. MDA, a secondary product of lipid peroxidation and a marker of oxidative damage rather than an antioxidant enzyme, was also elevated in exposed groups, indicating that the compensatory antioxidant response did not fully prevent membrane lipid peroxidation. Similarly, LDH and SDH, which are metabolic and tissue-damage markers rather than antioxidant enzymes, showed significant changes consistent with altered energy metabolism and cellular stress. Collectively, these results indicate that copper exposure induces oxidative stress and oxidative damage, but the antioxidant response is not a simple concentration-dependent upregulation of all enzymes; rather, it involves a coordinated but partially insufficient activation of specific antioxidant pathways.

The target of rapamycin (TOR), as a key nutrient-sensing regulator, promotes protein synthesis by activating S6 kinase (S6K) [26]. In juvenile carp (Catla), tryptophan upregulates TOR expression, thereby enhancing protein deposition and digestive enzyme activity [27]; this study revealed a ‘rise–fall–rise’ pattern in TOR expression at the transcriptional level. This pattern indicates a disruption in protein homeostasis during acute copper exposure. With the exception of the 300 μg/L group, transcriptional levels recovered in all groups following detoxification, suggesting the presence of persistent proteinotoxic stress requiring increased TOR expression for tissue repair.

Tumor necrosis factor-alpha (TNF-α) functions as a pivotal proinflammatory cytokine, activating NF-κB and JNK pathways to mediate inflammation, apoptosis, and antimicrobial defense [28]. Chronic TNF-α elevation (e.g., during bacterial infection) promotes hepatocyte apoptosis through reactive oxygen species (ROS) accumulation [29]. Here, sustained TNF-α upregulation during copper exposure followed by significant downregulation during depuration indicates immune activation potentially triggering inflammatory cascades and ROS-mediated apoptosis.

SREBP1 regulates the transcription of lipogenic genes such as FAS and ACCα, thereby driving the synthesis of fatty acids and triglycerides in the liver and increasing the area of lipid droplets [30]. The upregulation of SREBP1 in muscle during copper stress (which returns to baseline levels following copper clearance) may reflect a compensatory feedback mechanism for lipid metabolic dysfunction in muscle—a phenomenon that may be associated with the dysregulation of succinate dehydrogenase (SDH) observed in this study, although mechanistic validation remains required.

Fatty acid synthase (FAS) catalyzes saturated fatty acid production from acetyl-CoA and malonyl-CoA [31]. Its significant induction in muscle during exposure implies substantial saturated lipid synthesis, potentially compromising flesh quality. Leptin receptor (lp1) mediates appetite regulation and energy homeostasis via JAK2/STAT3 signaling, modulating lipolysis [32]. Elevated lp1 expression during exposure suggests abnormal intramuscular lipid accumulation consistent with FAS-mediated lipogenesis. In addition, antioxidant-related genes (aoc, cat-1α, hsp70, hsp90) exhibited significant upregulation during stress, further confirming systemic oxidative stress activation under copper challenge.

### 4.2. Copper Exposure Triggers Immunoproteomic Remodeling in Fish Muscle: Paradoxical Metal Loss and Protein Accumulation

Copper is known to preferentially accumulate in metabolically active organs such as the liver and kidney, which rely on copper-dependent enzymes for energy production, antioxidant defense, and depuration [33]. Muscle tissue, traditionally viewed as a non-target organ for metal accumulation, exhibits lower basal copper retention. Strikingly, in the present study, copper exposure led to a significant decrease in muscle copper content compared to both control and recovery groups—a paradoxical finding that suggests the activation of active metal efflux mechanisms during short-term stress. Concomitantly, total muscle protein content increased markedly. Because crude protein was measured on a wet-weight basis, the increase in B4 could reflect either increased protein deposition or reduced tissue hydration. After dry-matter normalization, the increase remained significant, indicating that hydration shifts alone do not explain the difference. However, because protein synthesis and degradation rates were not directly measured, we cannot conclude that de novo synthesis of immune proteins occurred. We therefore describe this as an increase in crude protein content that may involve enhanced protein synthesis and/or reduced protein degradation. These observations indicate that even a tissue with low baseline copper retention can mount a robust molecular response to metal stress, with potential implications for local immune function and systemic health.

### 4.3. From Oxidative Damage to Immunometabolic Dysregulation: Copper-Induced Remodeling of Rainbow Trout Muscle

The dynamic reprogramming of gene expression and metabolite profiles is a hallmark of the integrated stress response, and our time-series data reveal two distinct adaptive trajectories in rainbow trout muscle following copper challenge. The first pattern—characterized by an initial downregulation of 51 genes during exposure followed by significant upregulation during depuration—suggests a delayed activation of transcriptional programs involved in tissue repair and immune restoration. Notably, this pattern was mirrored by 261 metabolites (positive-ion mode) and 162 metabolites (negative-ion mode), indicating that metabolic recovery lags behind the removal of the toxicant. We interpret this coordinated re-expression of genes and metabolites as a signature of immunometabolic regeneration, potentially involving the clearance of damaged macromolecules, replenishment of energy stores, and resolution of inflammation [33]. The contrasting pattern—sustained upregulation of 51 genes during exposure followed by downregulation during recovery—was accompanied by a progressive decrease (Profile 1) of 249 (POS) and 179 (NEG) metabolites that stabilized during depuration. This suggests that an initial wave of transcriptional activation, likely aimed at countering oxidative stress and initiating innate immune signaling, is gradually resolved as copper burden diminishes. The parallel downregulation of metabolites points to a transient shift in cellular metabolism that prioritizes defense over growth. Importantly, the persistence of certain lipid related metabolites during recovery indicates that some aspects of metabolic reprogramming may outlast the acute stress period, potentially sensitizing the immune system to secondary challenges. Collectively, these temporal patterns illustrate that copper exposure triggers not only a transient acute response but also a prolonged phase of immune metabolic adaptation, with implications for the long term health and disease resistance of fish in aquaculture settings.

Joint analysis of the prioritized genes and metabolites identified 11 genes and 94 metabolites exhibiting significant correlations, indicating their pivotal roles in the overall process. These key genes were categorized into four functional groups. The myofiber integrity axis (MYH4, Trim63, actc1a): The strong correlation of these structural genes with specific metabolites (e.g., branched-chain amino acids, acylcarnitines) indicates that copper exposure actively remodels the contractile apparatus. Upregulation of the E3 ubiquitin ligase Trim63 together with downregulation of MYH4 is a classic signature of atrophy-induced by pro-inflammatory cytokines (e.g., TNF-α). This catabolic program may serve to mobilize amino acids for the synthesis of acute-phase proteins and antioxidants, but at the expense of muscle integrity, potentially impairing swimming performance and escape behavior—traits directly linked to survival under predator pressure [34,35,36]. The lipid signaling axis (FABP2, PNPLA2, SCD1): The correlation of these lipogenic genes with saturated fatty acids and other lipid mediators suggests that copper exposure drives a pro-inflammatory lipid shift. Sustained SCD1 expression during depuration may represent an attempt to restore membrane fluidity and resolve inflammation; however, the persistent accumulation of saturated fatty acids indicates incomplete resolution. Such a state can sensitize immune cells to subsequent challenges, establishing a vicious cycle of low-grade inflammation that could compromise long-term disease resistance [37,38]. The stress–proteostasis axis (Cebpd, fastkd2, odc1-a): These genes are central to the integrated stress response (ISR) and redox homeostasis. Cebpd is a transcription factor that bridges oxidative stress to inflammatory gene expression, while odc1-a (rate-limiting for polyamine synthesis) is essential for cell proliferation and tissue repair. Their correlation with metabolites such as L-aspartic acid and 3-dehydrocarnitine reveals a metabolic switch that prioritizes depuration and repair over routine protein turnover. This adaptive response is crucial for surviving acute stress, but if sustained, it may divert resources from growth and immune surveillance [39,40]. The regeneration axis (Myod2, Fhl1): The synergistic interaction between Myod2 (myoblast activation) and Fhl1 (fiber maturation) is fundamental for muscle regeneration [41,42]. Their correlation with nucleotide-related metabolites suggests that copper-induced tissue damage activates repair programs. However, the persistence of these transcriptional changes into the depuration phase indicates that regeneration remains incomplete, leaving the tissue in a state of regenerative vulnerability. Such a state may impair the muscle’s ability to serve as an immune-competent organ, as damaged fibers can become niches for persistent inflammation or secondary infection [41,42].

Collectively, these four axes are not independent; they intersect at key signaling nodes (FoxO, mTOR, PPAR) that regulate inflammation, autophagy, and energy metabolism. The gene–metabolite correlations provide a systems-level view of how copper stress transforms muscle from a purely contractile tissue into an active immune–metabolic interface, with implications for both individual fitness and population health in polluted aquatic environments.

Several plant-derived compounds were tentatively annotated in muscle, including salvianolic acid B, glucoraphanin/raphasnin, gossypetin, cepharanthine, limonin, pomiferin, and neoandrographolide. Because these compounds are unusual in fish muscle and were not verified with authentic standards, they are reported as tentative annotations and may originate from feed or environmental exposure. They were not used to support mechanistic conclusions. The biological interpretation therefore focuses on endogenous metabolites such as amino acids, fatty acids, acylcarnitines, and nucleotides. Under copper ion stress, reactive oxygen species (ROS) are generated via the Fenton reaction. These ROS directly attack and oxidize key cellular components, including myofibrillar proteins (e.g., actin, encoded by actc1a), membrane phospholipids, and mitochondria. This oxidative damage induces protein cross-linking, denaturation, and degradation; increases membrane permeability; and impairs mitochondrial energy production, leading to an insufficient energy supply [43]. Concurrently, copper stress and resultant oxidative damage activate inflammatory signaling pathways (e.g., NF-κB), triggering the release of pro-inflammatory cytokines (such as TNF-α, IL-1β, and IL-6) and inducing muscle proteolytic pathways (including the ubiquitin–proteasome system and autophagy) [44,45]. Collectively, these processes contribute to muscle atrophy and deterioration of textural quality.

A comprehensive analysis indicates that several key metabolites affecting rainbow trout muscle under copper ion stress are regulated via MYH4-mediated pathways. Specifically: glycine–proline–arginine slows down the degradation of the MYH4 protein, thereby maintaining muscle toughness [46]. Histidine-glutamic acid-lysine protects the ultrastructure of myofibrils and improves post-mortem meat tenderness [47]. Myristoyl-L-carnitine promotes fatty acid β-oxidation, compensates for impaired glycolysis, and alleviates stress-induced muscle dysfunction caused by energy depletion [48]. Anthocyanins [M+] inhibit lipid peroxidation cascades, maintain muscle membrane integrity, and reduce drip loss [49]. Maltose, acting as a rapid energy substrate, maintains glycolytic flux, preserves pre-slaughter ATP levels, delays the onset of rigor mortis, and enhances water-holding capacity [50].

In addition, other metabolites involved in rainbow trout muscle metabolism have been identified, such as 4,4′-methylenebis(2,6-tert-butylphenol), a potent free radical scavenger that inhibits lipid peroxidation and the oxidative cross-linking of myofibrillar proteins (such as actin). This compound interacts with the ODC1-A gene to reduce protein degradation and maintain muscle structural integrity [51]. L-aspartic acid participates in the malate–aspartate transport system to enhance mitochondrial ATP production and support depuration via the urea cycle. It acts synergistically with the ODC1-A gene to maintain energy supply, thereby mitigating pH decline and osmotic losses caused by glycolysis [52]. 3-Dehydrocarnitine (a carnitine derivative) promotes energy metabolism by regulating fatty acid β-oxidation. Furthermore, it chelates copper ions to mitigate toxicity and acts synergistically with the fastkd2 gene to prevent abnormal lipid deposition and energy depletion [53].

### 4.4. Copper Stress Is Associated with Immune–Metabolic Dysregulation Involving FoxO, PPAR and mTOR Signaling in Rainbow Trout Muscle

**Figure 5:**
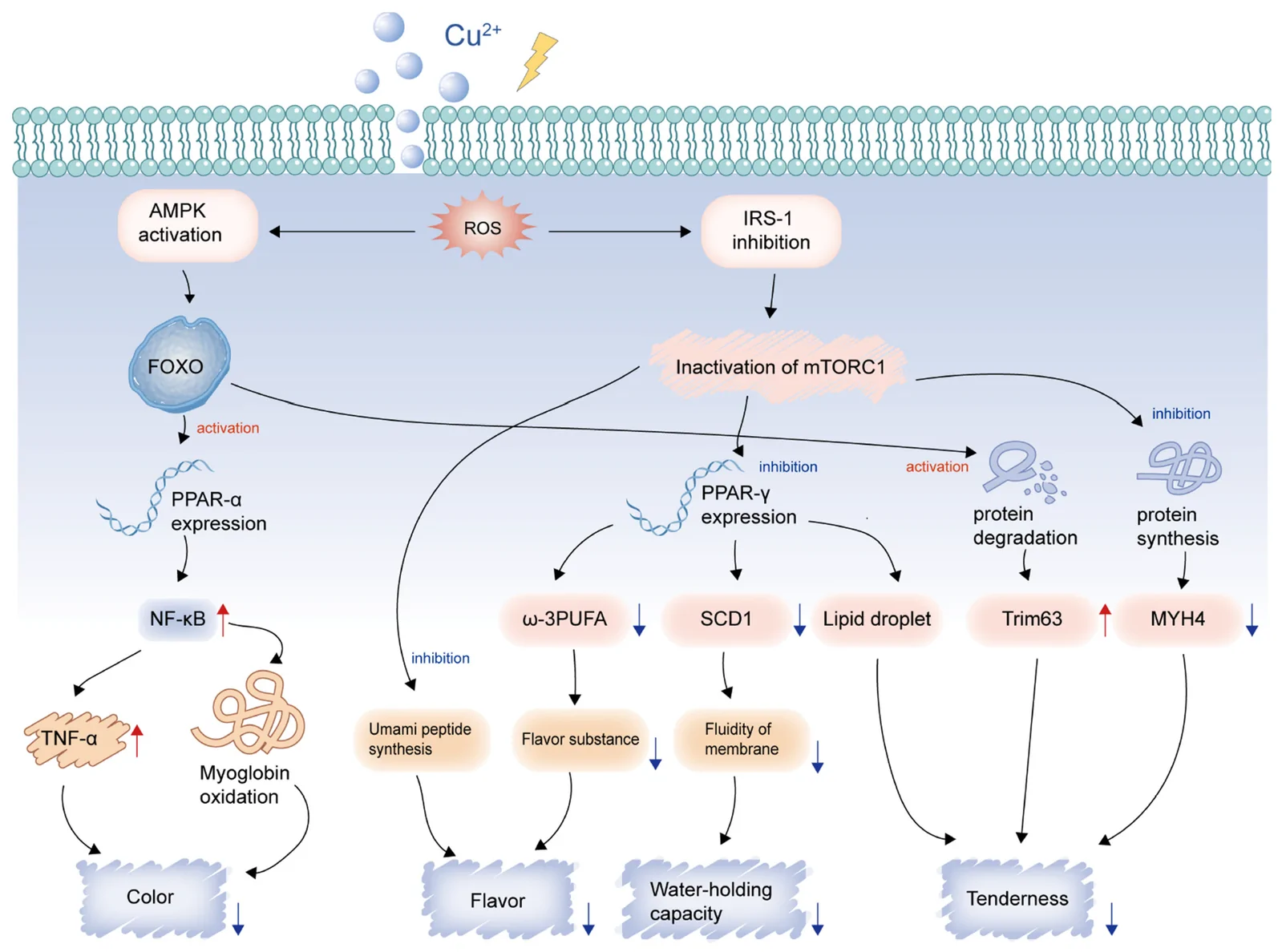
Potential regulatory mechanisms of copper ion stress on muscle quality in rainbow trout.

Genes enriched in the FoxO signaling pathway were differentially expressed and correlated with muscle immune and antioxidant parameters (Figure 5), suggesting a potential regulatory role in rainbow trout under copper stress by modulating protein metabolic homeostasis, antioxidant defense and energy metabolism [54]. Under physiological conditions, the insulin/insulin-like growth factor-1 (IGF-1) signaling pathway activates the PI3K-Akt cascade, inducing Akt-mediated phosphorylation of FoxO. This modification retains FoxO in the cytoplasm, thereby promoting protein synthesis (via mTOR activation) whilst inhibiting catabolic processes [55]. However, under copper ion stress, ROS-mediated inhibition of IRS-1 tyrosine phosphorylation impedes Akt activation. Dephosphorylated FoxO translocates to the nucleus and triggers two opposing responses: (1) It activates the transcription of CAT and SOD2, enhancing the clearance of copper-induced H_2_O_2_. This mitigates the oxidation of myoglobin [56]. (2) The sustained retention of FoxO in the nucleus promotes the upregulation of Trim63, thereby driving the ubiquitin-mediated degradation of myosin heavy chain (MYH4) [57,58].

The PPAR signaling pathway plays a key regulatory role in the muscle metabolism and inflammatory response of rainbow trout by modulating lipid homeostasis, energy metabolism and inflammatory responses [59]. We propose two mechanistic hypotheses: (1) Copper stress-induced inhibition of PPARα disrupts energy homeostasis. This energy deficit triggers protein degradation and the accumulation of misfolded proteins; (2) concomitant inactivation of PPARγ disrupts intramuscular lipid metabolism, promoting abnormal lipid deposition and increasing the content of saturated fatty acids (Figure 5).

The mTOR signaling pathway regulates protein synthesis, autophagy, energy metabolism, and lipogenesis in fish muscle [60]. In our transcriptomic dataset, Srebp1, FAS, and Scd1 were upregulated during copper exposure and declined during depuration, which is consistent with activation rather than suppression of the mTORC1–SREBP1 lipogenic axis. We therefore propose that copper stress is associated with putative activation of mTORC1–SREBP1 signaling, driving the transcription of FAS and SCD1 and promoting lipogenesis (Figure 5). The concurrent accumulation of saturated fatty acids in the metabolome suggests that this lipogenic activation is not matched by sufficient desaturation or β-oxidation capacity, resulting in a pro-inflammatory lipid profile.

## 5. Conclusions

This comprehensive multi-omics study demonstrates that short-term exposure to acute sublethal copper concentrations (75–300 µg/L) induces persistent changes in immune transcription levels in rainbow trout muscle. Copper stress triggered neurotoxicity (AChE inhibition), oxidative stress characterized by biphasic or dysregulated antioxidant enzyme activity (including persistent CAT suppression and non-monotonic T-SOD responses) and increased lipid peroxidation, and sustained upregulation of the pro-inflammatory cytokine TNF-α together with lipid metabolism genes (*Srebp1*, *FAS*), driving saturated fatty acid accumulation and chronic low-grade inflammation. Integrated network analysis identified *MYH4* and *SCD1* as central nodes, with FoxO, mTOR and PPAR pathways orchestrating immune-driven proteolysis, aberrant lipid signaling and energy dysregulation. However, this study also has certain limitations; the causal relationships of key gene–metabolite axes identified through multi-omics screening (such as *MYH4* and *SCD1*) have not yet been validated through gene knockdown, inhibitor experiments or protein validation. Furthermore, a 2-day recovery period is insufficient to assess irreversible toxicity; subsequent studies should employ a recovery period of 14 days or longer to distinguish between ‘delayed peaks’ and ‘persistent damage’.

## Figures and Tables

**Table 1:** RT-qPCR primer sequences for all target genes.

Gene	Sequence	Length
***TNF-α***	F: GGTTGGCTATGGAGGCTGTG R: TGCTTCAATGTATGGTGGGTA	170
***Lpl***	F: TAATTGGCTGCAGAAAACAC R: CGTCAGCAAACTCAAAGGT	174
***Fas***	F: TGATCTGAAGGCCCGTGTCA R: GGGTGACGTTGCCGTGGTAT	95
***Srebp1***	F: GACAAGGTGGTCCAGTTGCT R: CACACGTTAGTCCGCATCAC	97
***TOR***	F: CAGCCACACACTTTTACAGACC R: AATCTTGGTGAGGTACGGCTG	115
***Cat-1a***	F: TCGATTTTCAAGGGTCTTCG R: CACAACGATCAGCAAACTGG	159
***Aoc***	F: GCGCCAAGTACTTCCTCAAC R: TCACAAACTCCTGTGTGCTG	285
***HSP70***	F: TCATCACAGTCCCTGCCTAC R: CGGTTATCAAAGTCCTCCC	280
***HSP90***	F: CAATGACTGGGAGGAACA R: TCTCAAAGAGGTCGAAGG	110
***β-actin***	F: TGGGGCAGTATGGCTTGTATG R: CTCTGGCACCCTAATCACCTCT	165

**Table 2:** Overview of transcriptome data from nine libraries of rainbow trout muscle.

Item	T4-1	T4-2	T4-3	B4-1	B4-2	B4-3	B6-1	B6-2	B6-3
Raw data (bp)	7,861,062,300	6,354,532,200	6,506,389,500	6,923,860,800	5,756,247,300	5,759,715,900	5,776,299,000	5,506,656,000	5,464,464,300
Q30 (%)	95.67	95.71	95.72	94.22	95.58	95.70	95.59	95.42	95.52
Clean reads	52,093,658	42,099,198	43,117,000	45,848,026	38,135,294	38,187,136	38,251,514	36,453,842	36,203,044
Total mapped (%)	96.60	96.88	96.82	96.24	96.90	96.88	96.81	96.85	96.91
Exon (%)	93.81	94.40	94.63	94.44	94.56	94.09	94.18	94.04	94.84
Sequenced know genes	31,234	30,601	31,050	31,034	29,907	30,799	30,985	31,164	29,949
Sequenced novel genes	468	465	462	459	457	470	457	466	463
Sequenced total genes	31,702	31,066	31,512	31,493	30,364	31,269	31,442	31,630	30,412

## Data Availability

All raw transcriptome data reported and sample metadata expression estimates in this article have been deposited with the National Genomics Data Center (https://ngdc.cncb.ac.cn, accessed on 22 September 2025) under accession numbers CRA027418.

## Supplementary Materials

The following supporting information can be downloaded at https://www.mdpi.com/article/10.3390/biology15211913/s1, Supplementary File S1: Analysis methods of transcriptome bioinformatics; Supplementary File S2: Analysis methods of Metabolomic bioinformatics.

## References

[1] Isani G., Falcioni M.L., Barucca G., Sekar D., Andreani G., Carpenè E., Falcioni G. (2013). Comparative toxicity of CuO nanoparticles and CuSO_4_ in rainbow trout. Ecotoxicol. Environ. Saf..

[2] Lin J., Wang L., Huang M., Xu G., Yang M. (2024). Metabolic changes induced by heavy metal copper exposure in human ovarian granulosa cells. Ecotoxicol. Environ. Saf..

[3] Linder M.C. (2020). Copper Homeostasis in Mammals, with Emphasis on Secretion and Excretion. A Review. Int. J. Mol. Sci..

[4] Dong Y., Dong S., Yu C., Liu J., Gai S., Xie Y., Zhao Z., Qin X., Feng L., Yang P. (2023). Mitochondria-targeting Cu_3_VS_4_ nanostructure with high copper ionic mobility for photothermoelectric therapy. Sci. Adv..

[5] Lutsenko S., Roy S., Tsvetkov P. (2025). Mammalian copper homeostasis: Physiological roles and molecular mechanisms. Physiol. Rev..

[6] Li G., Liang Z., Sun J., Qiu Y., Qiu C., Liang X., Zhu Y., Wang P., Li Y., Jiang F. (2021). A pilot-scale sulfur-based sulfidogenic system for the treatment of Cu-laden electroplating wastewater using real domestic sewage as electron donor. Water Res..

[7] Pilehvar A., Cordery K.I., Town R.M., Blust R. (2020). The synergistic toxicity of Cd (II) and Cu (II) to zebrafish (*Danio rerio*): Effect of water hardness. Chemosphere.

[8] Liu H., Xu Y., Sun Y., Wu H., Hou J. (2024). Tissue-specific toxic effects of nano-copper on zebrafish. Environ. Res..

[9] Comber S., Deviller G., Wilson I., Peters A., Merrington G., Borrelli P., Baken S. (2023). Sources of copper into the European aquatic environment. Integr. Environ. Assess. Manag..

[10] (2007). Aquatic Life Ambient Freshwater Quality Criteria—Copper.

[11] Shan J., Tian X., Guan C., Zhang C., Zhang Y., Chen S. (2022). Effect of Copper Ion Sterilization on Bacterial Community in a Freshwater Recirculating Aquaculture System. Curr. Microbiol..

[12] Buley R.P., Gladfelter M.F., Fernandez-Figueroa E.G., Wilson A.E. (2023). Complex effects of dissolved organic matter, temperature, and initial bloom density on the efficacy of hydrogen peroxide to control cyanobacteria. Environ. Sci. Pollut. Res. Int..

[13] Tesser M.E., de Paula A.A., Risso W.E., Monteiro R.A., do Espirito Santo Pereira A., Fraceto L.F., Bueno Dos Reis Martinez C. (2020). Sublethal effects of waterborne copper and copper nanoparticles on the freshwater Neotropical teleost *Prochilodus lineatus*: A comparative approach. Sci. Total Environ..

[14] Bourdon C., Couture P., Gourves P.Y., Clérandeau C., Gonzalez P., Cachot J. (2023). Comparison of the accumulation and effects of copper pyrithione and copper sulphate on rainbow trout larvae. Environ. Toxicol. Pharmacol..

[15] Tsang T., Davis C.I., Brady D.C. (2021). Copper biology. Curr. Biol..

[16] Lu Y., Li L., Zhu Y., Wang X., Li M., Lin Z., Hu X., Zhang Y., Yin Q., Xia H. (2018). Multifunctional Copper-Containing Carboxymethyl Chitosan/Alginate Scaffolds for Eradicating Clinical Bacterial Infection and Promoting Bone Formation. ACS Appl. Mater. Interfaces.

[17] Zhu Z., Song M., Ren J., Liang L., Mao G., Chen M. (2024). Copper homeostasis and cuproptosis in central nervous system diseases. Cell Death Dis..

[18] Wang X.X., Chen W.Z., Li C., Xu R.S. (2024). Current potential pathogenic mechanisms of copper-zinc superoxide dismutase 1 (SOD1) in amyotrophic lateral sclerosis. Rev. Neurosci..

[19] Tang Q.Q., Feng L., Jiang W.D., Liu Y., Jiang J., Li S.H., Kuang S.Y., Tang L., Zhou X.Q. (2013). Effects of dietary copper on growth, digestive, and brush border enzyme activities and antioxidant defense of hepatopancreas and intestine for young grass carp (*Ctenopharyngodon idella*). Biol. Trace Elem. Res..

[20] Musharraf M., Khan M.A. (2022). Estimation of dietary copper requirement of fingerling Indian major carp, *Labeo rohita* (Hamilton). Aquaculture.

[21] Karalaki M., Fili S., Philippou A., Koutsilieris M. (2009). Muscle regeneration: Cellular and molecular events. In Vivo.

[22] Lu Y.L., Su J., Li J.W., Xu W.R. (2024). A molecular container providing supramolecular protection against acetylcholine hydrolysis. Org. Biomol. Chem..

[23] Xu H., Van Remmen H. (2021). The SarcoEndoplasmic Reticulum Calcium ATPase (SERCA) pump: A potential target for intervention in aging and skeletal muscle pathologies. Skelet. Muscle.

[24] Wang X., Zhang X., Cao K., Zeng M., Fu X., Zheng A., Zhang F., Gao F., Zou X., Li H. (2022). Cardiac disruption of SDHAF4-mediated mitochondrial complex II assembly promotes dilated cardiomyopathy. Nat. Commun..

[25] Zhang Y., Zhang S., Zhong L., Liu G., Sui Y., Wang M., Jiang H. (2025). The role of cortisol in the acute immune regulation of channel catfish (*Ictalurus punctatus*) spleen stimulated by *Aeromonas hydrophila*. Comp. Biochem. Physiol. Part D Genom. Proteom..

[26] González A., Hall M.N., Lin S.C., Hardie D.G. (2020). AMPK and TOR: The Yin and Yang of Cellular Nutrient Sensing and Growth Control. Cell Metab..

[27] Yasmin F., Sutradhar S., Roy A., Mukherjee S. (2025). Effects of dietary quality on melatonin in gut, serum and ovary in mediating growth, digestive and ovarian physiology in juvenile carp (*Catla catla*). Gen. Comp. Endocrinol..

[28] Jaldeep L., Lipi B., Prakash P. (2025). Neurotrophomodulatory effect of TNF-α through NF-κB in rat cortical astrocytes. Cytotechnology.

[29] Popescu M., Cabrera-Martinez B., Winslow G.M. (2019). TNF-α Contributes to Lymphoid Tissue Disorganization and Germinal Center B Cell Suppression during Intracellular Bacterial Infection. J. Immunol..

[30] Zhao Z., Xiang X., Chen Q., Du J., Zhu S., Xu X., Shen Y., Wen S., Li Y., Xu W. (2024). Sterol Regulatory Element Binding Protein 1: A Mediator for High-Fat Diet-Induced Hepatic Gluconeogenesis and Glucose Intolerance in Fish. J. Nutr..

[31] Campa D., McKay J., Sinilnikova O., Hüsing A., Vogel U., Hansen R.D., Overvad K., Witt P.M., Clavel-Chapelon F., Boutron-Ruault M.C. (2009). Genetic variation in genes of the fatty acid synthesis pathway and breast cancer risk. Breast Cancer Res. Treat..

[32] Tripathy S., Bhattamisra S.K. (2025). Cellular signalling of melatonin and its role in metabolic disorders. Mol. Biol. Rep..

[33] Lu J., Quan J., Zhou J., Liu Z., Ding J., Shang T., Zhao G., Li L., Zhao Y., Li X. (2024). Combined transcriptomics and metabolomics to reveal the effects of copper exposure on the liver of rainbow trout (*Oncorhynchus mykiss*). Ecotoxicol. Environ. Saf..

[34] Chardulo L.A.L., Baldassini W.A., Curi R.A., Pereira G.L., Machado Neto O.R., Dal-Pai M., Vechetti-Júnior I.J., Malheiros J.M., Enriquez-Valencia C.E. (2021). Gene and protein expression of myosin heavy chain in Nellore cattle comparing growth or meat tenderness traits. Anim. Biotechnol..

[35] Fonseca L.F.S., Gimenez D.F.J., Dos Santos Silva D.B., Barthelson R., Baldi F., Ferro J.A., Albuquerque L.G. (2017). Differences in global gene expression in muscle tissue of *Nellore cattle* with divergent meat tenderness. BMC Genom..

[36] Zhao L., Li F., Zhang X., Tian H., Ma Z., Yang X., Zhang Q., Pu M., Cao P., Zhang D. (2025). RNA-Seq and WGCNA Identify Key Regulatory Modules and Genes Associated with Water-Holding Capacity and Tenderness in Sheep. Animals.

[37] Sun J., Xie F., Wang J., Luo J., Chen T., Jiang Q., Xi Q., Liu G.E., Zhang Y. (2024). Integrated meta-omics reveals the regulatory landscape involved in lipid metabolism between pig breeds. Microbiome.

[38] Du J., Qin H. (2024). Lipid metabolism dynamics in cancer stem cells: Potential targets for cancers. Front. Pharmacol..

[39] Costa-Mattioli M., Walter P. (2020). The integrated stress response: From mechanism to disease. Science.

[40] Rojas-Morales P., Pedraza-Chaverri J., Tapia E. (2020). Ketone bodies, stress response, and redox homeostasis. Redox Biol..

[41] Chen S.L., Wu C.C., Li N., Weng T.H. (2024). Post-transcriptional regulation of myogenic transcription factors during muscle development and pathogenesis. J. Muscle Res. Cell Motil..

[42] Picard B., Gagaoua M., Al Jammas M., Bonnet M. (2019). Beef tenderness and intramuscular fat proteomic biomarkers: Effect of gender and rearing practices. J. Proteom..

[43] Choi H., Kim S., Kwak M., Gwak Y., Jeong K., Seo Y., Yoo D. (2024). Cu Anchored Carbon Nitride (Cu/CN) Catalyzes Selective Oxidation of Thiol by Controlling Reactive Oxygen Species Generation. ACS Nano.

[44] Wen H., Miao W., Liu B., Chen S., Zhang J.S., Chen C., Quan M.Y. (2024). SPAUTIN-1 alleviates LPS-induced acute lung injury by inhibiting NF-κB pathway in neutrophils. Int. Immunopharmacol..

[45] Guo L., Li Y., Li W., Qiu J., Du J., Wang L., Zhang T. (2022). Shikonin ameliorates oxidative stress and neuroinflammation via the Akt/ERK/JNK/NF-κB signalling pathways in a model of Parkinson’s disease. Clin. Exp. Pharmacol. Physiol..

[46] Paneru B.D., Al-Tobasei R., Kenney B., Leeds T.D., Salem M. (2017). RNA-Seq reveals MicroRNA expression signature and genetic polymorphism associated with growth and muscle quality traits in rainbow trout. Sci. Rep..

[47] Demirkale İ. (2023). Investigation of ultrastructure and texture changes of fillets of rainbow trout grown under different aquaculture systems. Cell. Mol. Biol..

[48] Enkler L., Szentgyörgyi V., Pennauer M., Prescianotto-Baschong C., Riezman I., Wiesyk A., Avraham R.E., Spiess M., Zalckvar E., Kucharczyk R. (2023). Arf1 coordinates fatty acid metabolism and mitochondrial homeostasis. Nat. Cell Biol..

[49] Hashemi S., Asrar Z., Pourseyedi S., Nadernejad N. (2019). Investigation of ZnO nanoparticles on proline, anthocyanin contents and photosynthetic pigments and lipid peroxidation in the soybean. IET Nanobiotechnol..

[50] Li J., Wang H., Liang R., Mao Y., Yang X., Luo X., Zhu L., Zhang Y. (2025). Effect of super-fast freezing before rigor mortis on quality of bovine *M. longissimus lumborum*. Meat Sci..

[51] Chandimali N., Bak S.G., Park E.H., Lim H.J., Won Y.S., Kim E.K., Park S.I., Lee S.J. (2025). Free radicals and their impact on health and antioxidant defenses: A review. Cell Death Discov..

[52] Ackermann T., Zuidhof H.R., Müller C., Kortman G., Rutten M.G.S., Broekhuis M.J.C., Zaini M.A., Hartleben G., Calkhoven C.F. (2023). C/EBPβ-LIP mediated activation of the malate-aspartate shuttle sensitizes cells to glycolysis inhibition. Mol. Metab..

[53] Wu Y., Fu W., Liu L., Jiang Y., Liu N., Fang M., Ye H., Li J., Chu Z., Qian H. (2024). APTES-mediated Cu_2_(OH)_3_(NO_3_) nanomaterials on the surface of silicone catheters for abscess. Colloids Surf. B Biointerfaces.

[54] Jiang W.D., Wen H.L., Liu Y., Jiang J., Wu P., Zhao J., Kuang S.Y., Tang L., Tang W.N., Zhang Y.A. (2016). Enhanced muscle nutrient content and flesh quality, resulting from tryptophan, is associated with anti-oxidative damage referred to the Nrf2 and TOR signalling factors in young grass carp (*Ctenopharyngodon idella*): Avoid tryptophan deficiency or excess. Food Chem..

[55] Glaviano A., Foo A.S.C., Lam H.Y., Yap K.C.H., Jacot W., Jones R.H., Eng H., Nair M.G., Makvandi P., Geoerger B. (2023). PI3K/AKT/mTOR signaling transduction pathway and targeted therapies in cancer. Mol. Cancer.

[56] Wang Q., Kuroda Y., Yang L., Lai S., Mizutani Y., Iddamalgoda A., Guo J., Yamamoto A., Murase D., Takahashi Y. (2021). GPNMB Extracellular Fragment Protects Melanocytes from Oxidative Stress by Inhibiting AKT Phosphorylation Independent of CD44. Int. J. Mol. Sci..

[57] Waddell D.S., Baehr L.M., van den Brandt J., Johnsen S.A., Reichardt H.M., Furlow J.D., Bodine S.C. (2008). The glucocorticoid receptor and FOXO1 synergistically activate the skeletal muscle atrophy-associated MuRF1 gene. Am. J. Physiol.-Endocrinol. Metab..

[58] Clarke B.A., Drujan D., Willis M.S., Murphy L.O., Corpina R.A., Burova E., Rakhilin S.V., Stitt T.N., Patterson C., Latres E. (2007). The E3 Ligase MuRF1 degrades myosin heavy chain protein in dexamethasone-treated skeletal muscle. Cell Metab..

[59] Zhang M., Li F., Ma X.F., Li W.T., Jiang R.R., Han R.L., Li G.X., Wang Y.B., Li Z.Y., Tian Y.D. (2019). Identification of differentially expressed genes and pathways between intramuscular and abdominal fat-derived preadipocyte differentiation of chickens in vitro. BMC Genom..

[60] Dai X., Li X., Yin D., Chen X., Wang L., Pang L., Fu Y. (2024). Identification and characterization of TOR in *Macrobrachium rosenbergii* and its role in muscle protein and lipid production. Sci. Rep..

