# Supplementary material for: Acute Copper Exposure Induces Immunotoxicity and Metabolic Reprogramming in Rainbow Trout (Oncorhynchus mykiss) Muscle via FoxO/mTOR/PPAR Pathways
Source: Biology (Basel). 2026 Oct 22;15(21):1913. doi: 10.3390/biology15211913 (PMC13761420; doi:10.3390/biology15211913)
Supplement: Supplementary file 1 [file biology-15-01913-s001.zip › Supplementary File S1.pdf]

S2.5. Bioinformatics analysis of transcriptome

S2.5.1. Bioinformatics analysis

Reads obtained from the sequencing machines includes raw reads containing adapters or low quality bases which will affect the following assembly and analysis. Thus, to get high quality clean reads, reads were further filtered by fastp (version 0.18.0). The parameters were as follows: (1) removing reads containing adapters; (2) removing reads containing more than 10% of unknown nucleotides(N); (3) removing low quality reads containing more than 50% of low quality (Q-value≤20) bases.

Short reads alignment tool Bowtie2 (version 2.2.8) was used for mapping reads to ribosome RNA (rRNA) database. The rRNA mapped reads then will be removed. The remaining clean reads were further used in assembly and gene abundance calculation.

S2.5.2. Alignment with Reference Genome

Based on the 2020 update of the rainbow trout reference genome USDA_OmykA_1.1 (https://www.ncbi.nlm.nih.gov/datasets/genome/GCF_013265735.2/), an index of the reference genome was created and paired-end clean sequences were mapped to the reference genome using HISAT2. parameters such as 2.4 were set to default values.

S2.5.3. Quantification of Gene Abundance

The mapped reads of each sample were assembled by using StringTie v1.3.1 in a reference-based approach. For each transcription region, a FPKM (fragment per kilobase of transcript per million mapped reads) value was calculated to quantify its expression abundance and variations, using RSEM software.

S2.5.4. Differentially expressed genes (DEGs)

RNAs differential expression analysis was performed by DESeq2 software between two different groups (and by edgeR between two samples). The genes/transcripts with the parameter of false discovery rate (FDR) below 0.05 and absolute fold change≥2 were considered differentially expressed genes/transcripts.

S2.5.5. GO and KEGG enrichment Analysis

Gene Ontology (GO) is an international standardized gene functional classification system which offers a dynamic-updated controlled vocabulary and a strictly defined concept to comprehensively describe properties of genes and their products in any organism. GO has three ontologies: molecular function, cellular component and biological process. The basic unit of GO is GO-term. Each GO-term belongs to a type of ontology. GO enrichment analysis provides all GO terms that significantly enriched in DEGs comparing to the genome background, and filter the DEGs that correspond to biological functions. Firstly all DEGs were mapped to GO terms in the Gene Ontology database (http://www.geneontology.org/), gene numbers were calculated for every term, significantly enriched GO terms in DEGs comparing to the genome background were defined by hypergeometric test.

Genes usually interact with each other to play roles in certain biological functions. Pathway-based analysis helps to further understand genes biological functions. KEGG is the major public pathway-related database. Pathway enrichment analysis identified significantly enriched metabolic pathways or signal transduction pathways in DEGs comparing with the whole genome background.

S2.5.6. Trend analysis

Gene expression pattern analysis is used to cluster genes of similar expression patterns for multiple samples (at least 3 in a specific time point, space, or treatment dose size order). To examine the expression pattern of DEGs, the expression data of each sample (in the order of treatment) were normalized to 0, log2 (v1/v0), log2(v2/v0), and then clustered by Short Time-series Expression Miner software (STEM).

The parameters were set as follows:

(1) Maximum Unit Change in Model Profiles between Time Points is 1;

(2) Maximum output profiles number is 20 (similar profiles will be merged;

(3) Minimum ratio of fold change of DEGs is no less than 2.0. The clustered profiles with p-value $\leq$0.05 were considered as significant profiles.

Then the DEGs in all or each profile were subjected to Gene Ontology (GO) and KEGG pathway enrichment analysis. Through the hypothesis test of the p-value calculation and FDR correction, the GO terms or Pathways with Qvalue$\leq$0.05 were defined as significant enriched GO terms or pathways.
