# Supplementary material for: Acute Copper Exposure Induces Immunotoxicity and Metabolic Reprogramming in Rainbow Trout (Oncorhynchus mykiss) Muscle via FoxO/mTOR/PPAR Pathways
Source: Biology (Basel). 2026 Oct 22;15(21):1913. doi: 10.3390/biology15211913 (PMC13761420; doi:10.3390/biology15211913)
Supplement: Supplementary file 1 [file biology-15-01913-s001.zip › Supplementary File S2.pdf]

S2.7. Bioinformatics analysis of metabolome

S2.7.1. Data quality control

Positive ion mode (POS) and negative ion mode (NEG) are both used to detect metabolites,which can make the metabolite coverage higher and the detection effect better. In the process of subsequent data analysis, the positive and negative ion models are analyzed separately.

QC samples are usually used for quality control when the study of metabolomics is based on mass spectrometry. Theoretically, QC samples are the same. But there will be systematic errors in the process of sample extraction, detection or analysis, which will lead to differences between QC samples. Principle component analysis (PCA) was performed by R language gmodels (v2.18.1). The more dense the distribution of QC is, the more reliable results is.

S2.7.2. Cluster analysis

S2.7.2.1. Hierarchical cluster analysis

A dendrogram is a diagram that shows the hierarchical relationship between objects. Hierarchical cluster analysis (HCA) was performed on the data and the resulting dendrogram was calculated using the average linkage method.

S2.7.2.2. Cluster heatmap analysis

Data were normalized by z-score and hierarchical clustered by R package pheatmap. The heatmap can display the expression patterns of metabolites in all samples.

S2.7.2.3. Correlation analysis of replicas

Variability of metabolite composition and abundance between samples can be

quantified by correlation data between samples. Correlation analysis was performed by R and drawing heatmap by R package pheatmap. The closer the correlation coefficient gets to 1, the higher the similarity of metabolic composition and abundance are between samples.

S2.7.3. Multivariate statistical analysis

S2.7.3.1. PCA analysis

For a preliminary visualization of differences between different groups of samples, the unsupervised dimensionality reduction method principal component analysis (PCA) was applied in all samples using R package models (http://www.r-project.org/). PCA is a statistical procedure that converts hundreds of thousands of correlated metabolites variables into a set of values of linearly uncorrelated variables called principal components.

S2.7.3.2. PLS-DA

Partial least squares discriminant analysis (PLS-DA) is a supervised dimensionality reduction method in which class memberships are coded in matrix form into Y to better distinguish the metabolomics profile of two groups by screening variables correlated to class memberships. PLS- DA was applied in comparison groups using R package ropls (http://www.r-project.org/).

S2.7.3.3. OPLS-DA

Orthogonal projection to latent structures-discriminant analysis (OPLS-DA) is an extension of PLS-DA which incorporates an Orthogonal Signal Correction (OSC) filter into a PLS model. The basic concept in OPLS is to separate the systematic variation in

X into two parts, one that is correlated to Y and one that is not correlated (orthogonal) with Y. Only the Y- predictive variation is used to model the data. OPLS-DA was applied in comparison groups using R package models (http://www.r-project.org/).

The OPLS-DA model was further validated by cross-validation and permutation test. For cross-validation, the data was partitioned into seven subsets, where each of the subsets was then used as a validation set. R2 indicated the total variation in the data matrix that was explained by the model. Predictive ability (Q2) values represented the most recognized diagnostic statistical parameter to validate the OPLS-DA model in metabolomics.

Acceptable predictive model is considered for Q2 value greater than 0.4. Good predictive model is considered for Q2 value greater than 0.9. Permutation test randomly permutes class labels for 200 times and then produces a distribution of R2' values and Q2' values. In essence, a reliable model should yield significantly larger R2 and Q2 value compared to R2' and Q2' values generated from random models using the same data set.

S2.7.4. Differential metabolites analysis

S2.7.4.1. Differential metabolites identify

A variable importance in projection (VIP) score of (O)PLS model was applied to rank the metabolites that best distinguished between two groups. The threshold of VIP was set to 1. In addition, T-test was also used as a univariate analysis for screening differential metabolites. Those with a p value of T test < 0.05 and VIP$\geq$1 were considered differential metabolites between two groups.

S2.7.4.2. Differential metabolites volcano analysis

To understand the regulation of differential metabolites, the fold changes of abundance between two groups were futher calculated to draw the volcano plot.

S2.7.4.3. Differential metabolites VIP statistics

The abundance of differential metabolites in the same group was normalized by z-score. And then,the VIP score of OPLS-DA was used to draw the graph. The top 15 metabolites are shown in the variable importance in projection (VIP) score plot in descending order.

S2.7.4.4. Differential metabolites correlation heatmap

Pearson correlation coefficient and *p*-value were calculated by R function, cor. and cor.test. The closer the correlation coefficient gets to 1, the higher the similarity of metabolic abundance are. Correlations were called significant when $p \leq 0.05$. And correlation heatmap was drawn by R corrplot package.

S2.7.4.5. Differential metabolites cluster heatmap

The abundances of differential metabolites were normalized by z-score and hierarchical clustered by R package pheatmap to show the accumulation differences between two groups.

S2.7.4.6. Differential metabolites z-score analysis

Z-score (standard fraction) is based on the relative content conversion of metabolites, which is used to measure the relative content of metabolites at the same level. The calculating formula is as follows:

$z = (x - \mu) / \sigma.$

Here x is the abundance of metabolite, μ is the mean of the abundance of metabolite in every samples, σ is the standard deviation.

S2.7.4.7. Differential metabolites ROC analysis

ROC (Receiver Operating Characteristic) curve analysis by R pROC package to evaluate the predictive power of each of the discriminant metabolites. The cutpoint was determined for each biomarker by searching for those that yielded both high sensitivity and specificity. ROC curves were then plotted on the basis of the set of optimal sensitivity and specificity values. Area under the curve (AUC) was computed via numerical integration of the ROC curves. The metabolite signature that has the largest area under the ROC curve was identified as having the strongest predictive power for discrimating two groups.

The ROC curve was analyzed using R package pROC according to the significant different metabolites between two groups.

S2.7.5. Pathway analysis

S2.7.5.1. KEGG annotations and enrichment analysis of differential expressed metabolites

Kyoto Encyclopedia of Genes and Genomes (KEGG) is the major public pathway-related database that includes not only genes but metabolites. Metabolites were mapped to KEGG metabolic pathways for annotation and enrichment analysis. Pathway enrichment analysis identified significantly enriched metabolic pathways or signal transduction pathways in differential metabolites comparing with the whole background.

S2.7.5.2. MSEA

Metabolic Set Enrichment Analysis (MSEA) was also used to evaluate for pathway over-representation using the MetaboAnalyst module. The Small Molecule Pathway Database (SMPDB) library was used for the analysis, and global test for the quantitative enrichment analysis (QEA) by R package MSEAp.

S2.7.5.3. MetPA Analysis

The Pathway Analysis (https://www.metaboanalyst.ca/faces/home.xhtml) module combines results from powerful pathway enrichment analysis with pathway topology analysis to help researchers identify the most relevant pathways involved in the conditions under study. There are 15 pathway libraries currently supported, with a total of 1173 pathways was used for the analysis. During the analysis, betweenness centrality and Hypergeometric test was used for the powerful pathway enrichment analysis and pathway topology analysis. MetPA analysis was carried out using the different metabolites in the comparison group.

S2.7.6. Trend analysis

Metabolites expression pattern analysis is used to cluster metabolites of similar expression patterns for multiple samples (at least 3 in a specific time point, space, or treatment dose size order). To examine the expression pattern of all annotated metabolites, the expression data of each sample (in the order of treatment) were normalized to 0, log2 (v1/v0), log2(v2/v0), and then clustered by Short Time-series Expression Miner software (STEM, version 1.3.11).

The parameters were set as follows:

1) Maximum Unit Change in Model Profiles between Time Points is 1;

2) Maximum output profiles number is 20 (similar profiles will be merged).

The clustered profiles with *p*-value ≤0.05 were considered as significant profiles. Then the metabolites in all or each profile were subjected to KEGG pathway enrichment analysis. Through the hypothesis test of the p-value calculation and FDR correction, the Pathways with Qvalue≤0.05 were defined as significant enriched pathways
